# Systemic immune response in young and elderly patients after traumatic brain injury

**DOI:** 10.1186/s12979-023-00369-1

**Published:** 2023-08-12

**Authors:** Marta Magatti, Francesca Pischiutta, Fabrizio Ortolano, Anna Pasotti, Enrico Caruso, Anna Cargnoni, Andrea Papait, Franco Capuzzi, Tommaso Zoerle, Marco Carbonara, Nino Stocchetti, Stefano Borsa, Marco Locatelli, Elisa Erba, Daniele Prati, Antonietta R Silini, Elisa R Zanier, Ornella Parolini

**Affiliations:** 1 Centro di Ricerca E. Menni, Fondazione Poliambulanza Istituto Ospedaliero, Brescia, Italy; 2 Department of Acute Brain Injury, Istituto di Ricerche Farmacologiche Mario Negri IRCCS, Milano, Italy; 3 Dipartimento di Anestesia-Rianimazione e Emergenza Urgenza, Fondazione IRCCS Ca’ Granda Ospedale Maggiore Policlinico, Milano, Italy; 4 Scienze della Vita e Sanità Pubblica, Università Cattolica del Sacro Cuore Facoltà di Medicina e Chirurgia, Roma, Italy; 5 Dipartimento Medicina di Laboratorio, Fondazione Poliambulanza Istituto Ospedaliero, Brescia, Italy; 6 Department of Pathophysiology and Transplantation, University of Milan, Milano, Italy; 7 Unit of Neurosurgery, Fondazione IRCCS Ca’ Granda Ospedale Maggiore Policlinico, Milano, Italy; 8 Department of Transfusion Medicine and Hematology, Fondazione IRCCS Ca’ Granda Ospedale Maggiore Policlinico, Milano, Italy; 9 Fondazione Policlinico Universitario “Agostino Gemelli” IRCCS, Roma, Italy

**Keywords:** Aging, Immune cells, Systemic immune cells, Traumatic brain injury, Elderly, Young

## Abstract

**Background:**

Traumatic brain injury (TBI) is a leading cause of death and long-term disability worldwide. In addition to primary brain damage, systemic immune alterations occur, with evidence for dysregulated immune responses in aggravating TBI outcome and complications. However, immune dysfunction following TBI has been only partially understood, especially in the elderly who represent a substantial proportion of TBI patients and worst outcome. Therefore, we aimed to conduct an in-depth immunological characterization of TBI patients, by evaluating both adaptive (T and B lymphocytes) and innate (NK and monocytes) immune cells of peripheral blood mononuclear cells (PBMC) collected acutely (< 48 h) after TBI in young (18–45 yo) and elderly (> 65 yo) patients, compared to age-matched controls, and also the levels of inflammatory biomarkers.

**Results:**

Our data show that young respond differently than elderly to TBI, highlighting the immune unfavourable status of elderly compared to young patients. While in young only CD4 T lymphocytes are activated by TBI, in elderly both CD4 and CD8 T cells are affected, and are induced to differentiate into subtypes with low cytotoxic activity, such as central memory CD4 T cells and memory precursor effector CD8 T cells. Moreover, TBI enhances the frequency of subsets that have not been previously investigated in TBI, namely the double negative CD27- IgD- and CD38-CD24- B lymphocytes, and CD56dim CD16- NK cells, both in young and elderly patients. TBI reduces the production of pro-inflammatory cytokines TNF-α and IL-6, and the expression of HLA-DM, HLA-DR, CD86/B7-2 in monocytes, suggesting a compromised ability to drive a pro-inflammatory response and to efficiently act as antigen presenting cells.

**Conclusions:**

We described the acute immunological response induced by TBI and its relation with injury severity, which could contribute to pathologic evolution and possibly outcome. The focus on age-related immunological differences could help design specific therapeutic interventions based on patients’ characteristics.

**Supplementary Information:**

The online version contains supplementary material available at 10.1186/s12979-023-00369-1.

## Background

Incidence of traumatic brain injury (TBI) is increasing worldwide with an estimation of 69 million cases each year and, despite advances in treatments, it remains a major cause of death and long-term disability [1–3]. In addition to the primary brain damage, secondary brain injuries contribute to the aggravation of TBI sequelae, with multiple organ failure and infections as the most frequent extracranial complications [4, 5]. Increasing evidence points to dysregulated systemic immune responses as a key element in aggravating TBI complications, and targeting aspects of the immune response may offer an effective strategy to treat TBI [6, 7]. Indeed, animal studies have shown significant improvement of TBI outcome after ghrelin-attenuation of leukocyte recruitment [8] or after monocyte depletion [9], and reduced edema formation, weakening of brain tissue loss, and decreased number of activated microglia/macrophages after neutrophil depletion [10]. In human patients, immune defects have been described mainly related to blood serum, such as acute and persistent increase of IgG and IgM auto-antibodies [11], elevated levels of complement factors C3 and C4, and altered levels of diverse cytokines including IL-4, IL-6, IL-8, and L-10 [12, 13]. Moreover, a decreased number of T lymphocytes, and the impairment of T- and natural killer (NK) cytotoxic activity has been observed in TBI patients [13–18]. However, TBI-induced immune cell dysfunction in patients remains poorly studied and only partially understood [6].

TBI epidemiology has changed in the last decades, and TBI rates in the elderly population are quickly rising, indeed the highest frequency of hospital admissions was recorded for patients over 65 years of age [19]. Immunological studies on the elderly have lagged behind the observations made in the young, but the former are increasingly susceptible to TBI, principally in high-income countries [20], with a mortality rate more than double that of young adults [21] and, in the survivor, quality of life is extremely affected [22, 23]. Moreover, during aging, the immune system undergoes a gradual time-dependent functional decline called immune senescence. This condition is characterized by cells with a higher susceptibility to apoptosis, reduced proliferative capacity, and an elevated production of inflammatory mediators that contribute to the creation of a low-grade but chronic, pro-inflammatory basal state. One of the most consistent alterations in a senescent immune system concerns the T cell compartment, which becomes characterized by a decreased thymic output of naïve T-cells and a relative increase of antigen-experienced memory T-cells [24]. NK cells shift from a less-mature CD56bright CD16+ subset to a mature CD56dim CD16+ subset, both of which possess reduced cytotoxicity activity [25]. Monocytes also undergo age-related changes characterized by an altered production of inflammatory cytokines, diminished responsiveness to ligands of the innate immune system, and transcriptional alterations of genes associated with defence mechanisms against bacteria and viruses [26, 27]. These age-related modifications could affect immune cell activation after TBI, with important contribution on pathology evolution and clinical outcome. However, studies investigating systemic immune alterations after TBI in adults included patients with a wide age range, from 18 to over 65 years, and no net distinction between young and elderly populations has been clearly performed [14, 28–30].

In this study, we aimed to give a comprehensive and in-depth overview of the age-related immunological changes occurring acutely in TBI patients. To this end, we conducted an immunological characterization of human peripheral blood mononuclear cells (PBMC) collected within 48 h from TBI in young (18–45 yo) and elderly (> 65 yo) patients. We first quantified blood biomarkers of neuronal damage and inflammation in relation to age and injury severity; we then performed a comprehensive characterization of immune cell subsets related to both adaptive (T and B lymphocytes) and innate immunity (NK cells and monocytes), and their diverse functional activity in terms of state of differentiation and cytokine production. Importantly, PBMC from TBI patients were compared to PBMC from age-matched controls in order to discriminate the systemic immune dysfunctions derived by TBI from those merely related to the immune senescence alterations typical of elderly patients.

## Results

### Subject characteristics

**Fig. 1:**
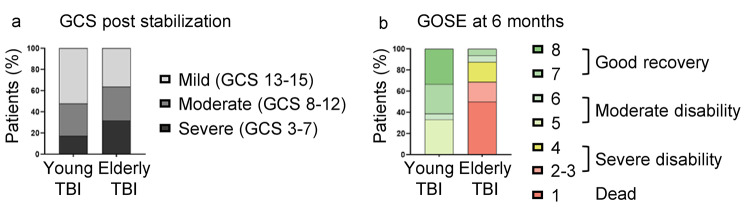
Patients’ characteristics. Percentage of TBI patients according to injury severity, assessed by Glasgow Coma Scale (GCS) post stabilization (**a**), and to 6-month outcome, assessed by Glasgow Outcome Scale Extended (GOSE) (**b**)

A total of 45 TBI patients were included, 23 of which were young adults (18-45yo) and 22 elderly (> 65yo), evaluated in the acute phase after TBI (Table 1). Injury severity of included patients, assessed by Glasgow Coma Scale (GCS) post stabilization, showed no significant differences across ages (Fig. 1a; Table 1). Instead, compared to young, elderly TBI patients showed higher mortality rate (p < 0.01, Table 1), and a worst 6-month outcome, assessed by Glasgow Outcome Scale Extended (GOSE, p < 0.001, Fig. 1b; Table 1), in accordance with TBI epidemiological data showing higher susceptibility to TBI damage in the elderly population [20].

**Table 1:** Subjects’ characteristics

	Young control	Young TBI	Elderly control	Elderly TBI	P
**Cohort size**, n	22	23	21	22	
**Age (year)**, Median (IQR)	32 (26–36)	27 (23–34)	77 (71–81)	79 (72–84)	***
**Sex (male)**, n (%)	9 (41)	14 (61)	12 (57)	13 (59)	
**GCS post stabilization**, Median (IQR)		10 (8.5–14)		9.5 (6.5–14)	
**Mild TBI** (GCS 13–15), n		12		8	
**Moderate TBI** (GCS 8–12), n		7		7	
**Severe TBI** (GCS 3–7), n		4		7	
**Pupils’ abnormalities**, n (%)		2 (9)		8 (36)	
**Subjected to surgery**, n (%)		5 (22)		9 (41)	
**Politrauma**, n (%)		8 (35)		4 (18)	
**Time to blood specimen collection (h from TBI)**, Median (IQR)		20 (14.5–30)		18 (14–24)	
**In hospital mortality**, n (%)		0 (0)		6 (27)	**
**Length of hospitalization (days)**, Median (IQR)		11 (4–22)		13.5 (6–23)	
**Length of hospitalization for patients who survived to hospital discharge (days)**, Median (IQR)		11 (4–22)		16.5 (11.6–26)	
**GCS at discharge (survivors)**, Median (IQR)		15 (15–15)		13.5 (11–15)	
**GOSE at 6 months available on n. of patients**		18/23		16/22	
**GOSE**, Median (IQR)		7 (5–8)		2 (1–4)	***
**Favourable outcome** (GOSE 5–8) (%)		18/18 (100)		2/16 (13)	***
**Unfavourable outcome** (GOSE 1–4) (%)		0/18 (0)		14/16 (87)	***

### Blood biomarkers of brain damage and inflammatory cytokines

**Fig. 2:**
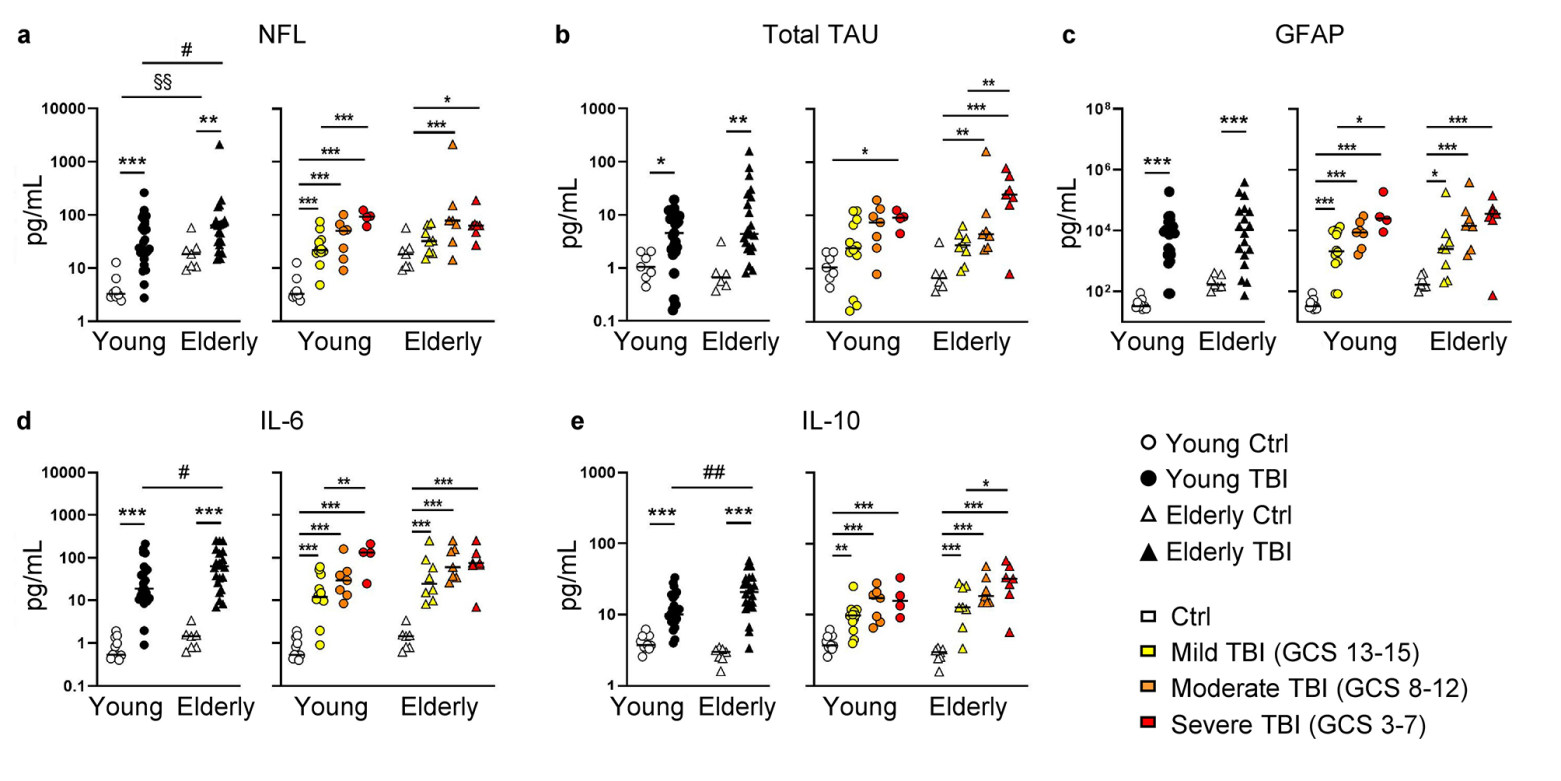
Blood biomarkers. Plasmatic levels of NfL (**a**), Total Tau (**b**), GFAP (**c**), IL-6 (**d**), and IL-10 (**e**) measured in plasma of young (circles) and elderly (triangles) donors. Left panels report data from healthy controls (white symbols) and TBI patients (black symbols). Right panels report data from healthy controls and TBI patients separated according to injury severity, i.e. mild (yellow), moderate (orange), and severe (red) TBI assessed as GCS score. * p < 0.05; ** p < 0.01; *** p < 0.001 TBI vs. control aged-matched subjects. §§ p < 0.01 young vs. elderly control subjects. # p < 0.05; ## p < 0.01 young vs. elderly TBI patients

Young and elderly TBI patients showed increased plasmatic levels of neuronal (NfL, Tau, Fig. 2a-b, left panel), astrocytic (GFAP, Fig. 2c, left panel) and inflammatory (IL-6, IL-10, Fig. 2d-e, left panel) markers compared to age-matched controls (See Supplementary Tables 1, Additional File 1). In addition, separating TBI patients according to injury severity (mild, moderate, and severe TBI, right panels), all markers displayed an injury-severity dependency, with severe TBI patients showing the highest amounts in both young and elderly populations, even if these effects were not statistically significant.

An age effect on NfL plasmatic levels was observed, with elderly controls and TBI patients showing higher levels of NfL than young subjects (Fig. 2a, left panel). Thus, TBI in elderly population results in additive susceptibility to neuronal damage. Inflammatory cytokines IL-6 and IL-10 did not show age-related differences in controls, however elderly TBI patients showed higher plasmatic levels compared to the young patients (Fig. 2d-e, left panel).

### Alterations in lymphoid and myeloid cell populations

**Fig. 3:**
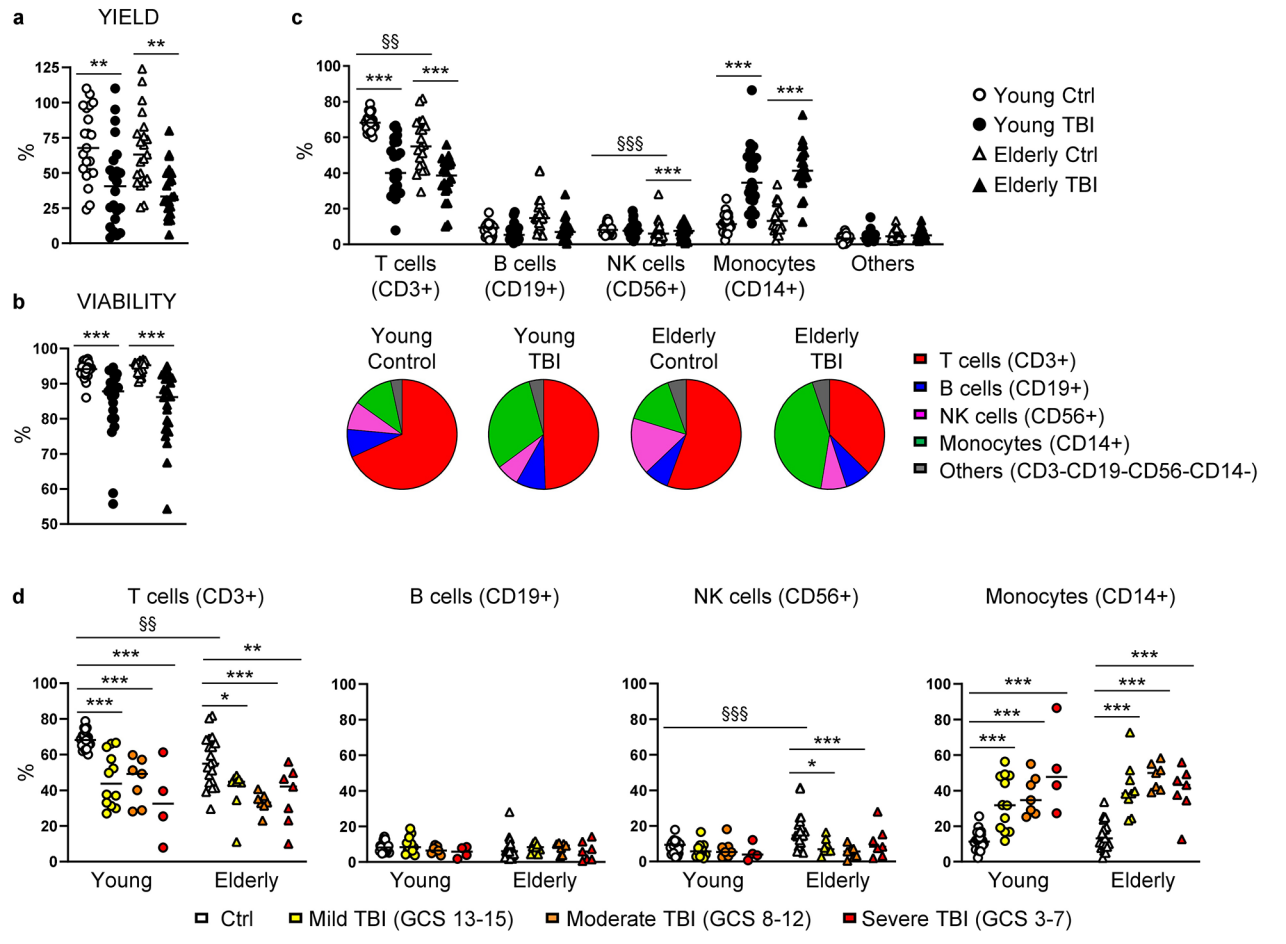
Analysis of lymphoid and myeloid cell populations. Percentage of yield (**a**) and viability (**b**) after thawing of PBMC from young (circles) and elderly (triangles) donors, control (white symbols) and TBI patients (black symbols). (**c**) Percentage of different immune subsets evaluated by flow cytometry. T cells were identified as CD3+, B cells as CD19+, NK cells as CD56+, and monocytes as CD14+ cells, in the morphological gate, after exclusion of dead cells and doublets. Pie chart of the mean of the percentage of different immune subsets was shown. (**d**) Results displayed in panel (**c**) are shown by separating TBI patients according to injury severity assessed as GCS score. * p < 0.05; ** p < 0.01; *** p < 0.001 TBI vs. control aged-matched subjects. §§ p < 0.01; §§§ p < 0.001 young vs. elderly control subjects

We obtained a significantly lower yield and cell viability after thawing PBMC from TBI patients when compared to age-matched controls (Fig. 3a-b), very likely indicating a higher susceptibility to cell death.

We first studied the frequencies of the main lymphoid and myeloid cell populations, including T cells (identified as CD3+ cells), B cells (CD19+), NK cells (CD56+), and monocytes (CD14+). Compared to healthy donors, TBI induced a significant reduction in the frequency of T cells in both young and elderly patients (Fig. 3c), and the reduction of T lymphocytes was not affected by trauma severity (Fig. 3d). No differences were observed in the frequency of CD19+ B cells (Fig. 3c). In addition, elderly TBI patients showed a reduced frequency of NK cells compared to aged-matched controls, while no differences were observed in young TBI patients (Fig. 3c). Finally, monocytes significantly increased in both young and elderly TBI patients (Fig. 3c), and the increment in TBI patients was not affected by trauma severity (Fig. 3d).

Next, to unravel the complex immunological changes caused by the trauma, we analysed the principal immune cell subsets of each immune population mentioned above, specifically of CD4 and CD8 T cells, B cells, NK cells, and monocytes (See Supplementary Tables 2, Additional File 2).

### Alterations in T lymphocyte subsets

**Fig. 4:**
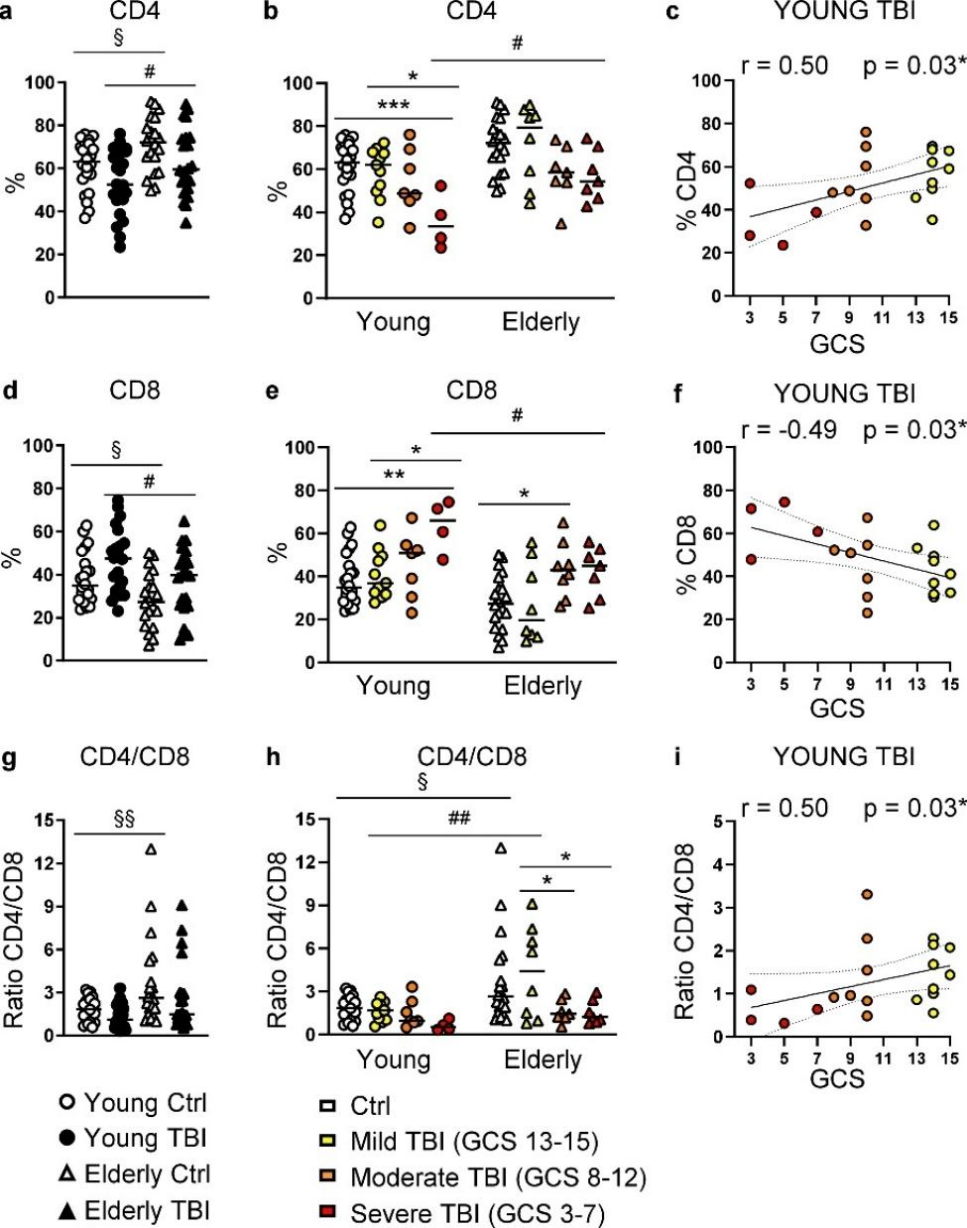
Analysis of T lymphocyte subsets. Percentage of CD4 T cells (identified in the gate of CD3+ T cells as CD4+) (**a**), CD8 T cells (identified as CD3+ CD4- T cells) (**d**), and the ratio of the percentage of CD4 and CD8 T cells (**g**) were shown in PBMC from young (circles) and elderly (triangles) donors, control (white symbols) and TBI patients (black symbols). Percentage of CD4 (**b**) and CD8 (**e**) T cells and the ratio CD4/CD8 T cells (**h**) were displayed by separating TBI patients according to injury severity assessed as GCS score. GCS score was correlated with the percentages of CD4 (**c**), CD8 T cells (**f**), and with the ratio CD4/CD8 (**i**) in young TBI patients. Correlations were assessed by Spearman correlation coefficients. Dashed lines represent 95% confidence intervals. Statistical significance is shown as p and r values. * p < 0.05; ** p < 0.01; *** p < 0.001 TBI vs. control aged-matched subjects. §§ p < 0.01; §§§ p < 0.001 young vs. elderly control subjects

T cells mature and develop into two main cell lineages: CD4 and CD8 T cells. In healthy subjects the frequency of CD4 is higher than CD8, and during aging the percentage of CD4 T cells increases (Fig. 4a) and that of CD8 T cells decreases (Fig. 4d). TBI altered the proportion of systemic CD4 and CD8 T lymphocytes, with a trend in the reduction of the frequency of CD4 (Fig. 4a) and an increase of CD8 (Fig. 4d) T cells, in both young and elderly TBI patients. A clear, injury-dependent effect was observed in young TBI patients where severe young patients had a statistically significant difference in the percentage of CD4 (Fig. 4b) and CD8 (Fig. 4e) T cells compared to healthy, age-matched controls. Confirming the injury-dependent effect, young TBI patients showed a linear correlation of GCS score with the percentage of CD4 (Fig. 4c) or CD8 (Fig. 4f). Such relation was less clear in elderly TBI patients.

Although not significant, the CD4/CD8 ratio after TBI was lower in both young (median 1.84 vs. 1.10, control vs. TBI, respectively) and elderly cohorts (median 2.64 vs. 1.50, control vs. TBI) (Fig. 4g, Supplementary Tables 2, Additional File 2). In the elderly, severe and moderate TBI patients showed the major CD4/CD8 ratio reduction when compared to mild patients (Fig. 4h). An injury-dependent effect was also observed in young patients who showed a linear correlation of GCS with the CD4/CD8 ratio (Fig. 4i).

**Fig. 5:**
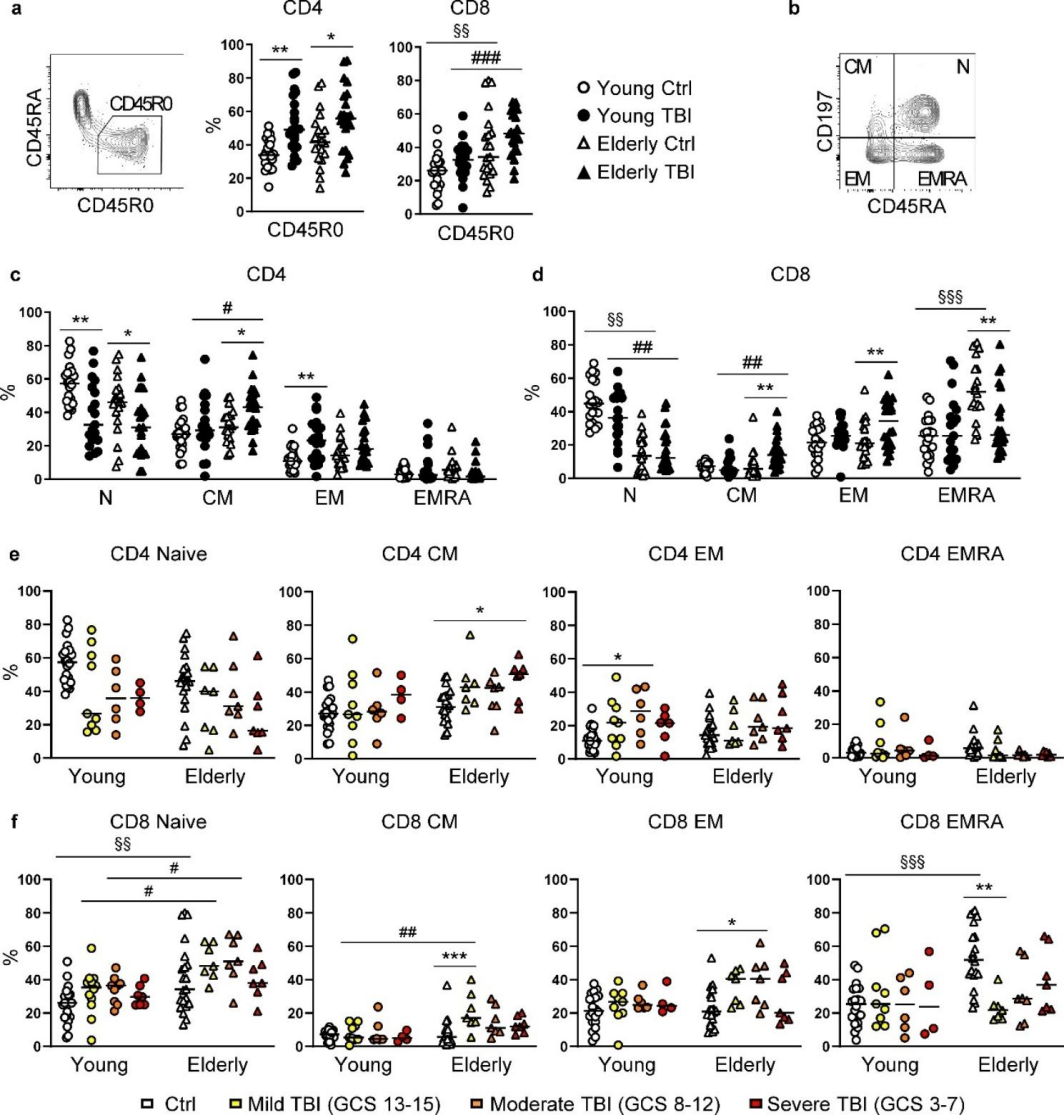
Analysis of T cell activation. (**a**) Percentage of activated CD4 and CD8 T cells was shown in PBMC from young (circles) and elderly (triangles) donors, control (white symbols) and TBI patients (black symbols). Activated T cells were identified as CD45R0+ CD45RA- in the gate CD3+ CD4+ for CD4 or CD3+ CD4- for CD8. (**b**) CD45RA and CD197/CCR7 markers were used to identified naïve (N), central memory (CM), effector memory (EM), and terminally differentiated effector memory (EMRA) T cells as follows. N: CD45RA+ CCR7+; CM: CD45RA- CCR7+; EM: CD45RA- CCR7-; EMRA: CD45RA+ CCR7-. The percentage of N, CM, EM, EMRA for both CD4 (**c**) and CD8 (**d**) T cells was shown in PBMC from young (circles) and elderly (triangles) donors, control (white symbols) and TBI patients (black symbols). The percentage of N, CM, EM, EMRA for CD4 (**e**) and CD8 (**f**) T cells was also analysed for injury severity, assessed as GCS. * p < 0.05; ** p < 0.01; *** p < 0.001 TBI vs. control aged-matched subjects. §§ p < 0.01; §§§ p < 0.001 young vs. elderly control subjects. # p < 0.05; ## p < 0.01; ### p < 0.001 young vs. elderly TBI patients

After T-cell stimulation there is a transition from a naïve CD45RA+ to an activated CD45R0 + phenotype [31] therefore in order to identify activated T cells, we analyzed the expression of CD45R0. TBI induced a significant increase of activated (CD45R0+) CD4 T cells in both young and elderly patients (Fig. 5a). During aging the frequency of activated (CD45R0+) CD8 T cells increased, both in healthy controls and TBI patients (Fig. 5a). Compared to respective age-matched controls, a minimal activation of CD8 lymphocytes was observed in young TBI patients (median 26.0 vs. 32.4, control vs. TBI, not significant), there was instead a more evident increase in elderly patients (median 34.2 vs. 48.2, control vs. TBI, not significant), suggesting a differential CD8 lymphocyte response between young and elderly patients after TBI (Fig. 5a).

Following activation, naïve T cells start to proliferate and differentiate into effector and memory precursors T cells, which develop into long-lived memory T cells. To date, a well appreciated model to define differentiation stages is based on the expression of the leukocyte common antigen isoform CD45RA and the chemokine receptor CCR7 (CD197) marker [32, 33]. Based on these markers, we analyzed the percentage of naïve (N; CD45RA+ CCR7+), central memory (CM; CD45RA- CCR7+), effector memory (EM; CD45RA- CCR7-), and terminally differentiated effector memory (EMRA; CD45RA+ CCR7-) CD4 and CD8 T cells (Fig. 5b). One of the most significant hallmarks of T-cell aging is the drop of naïve T cells and the increase of terminally differentiated T cells [34]. In line with this we observed the decline of naive T cells and the increase of EMRA cells with age in healthy control subjects, and these alterations were modest for CD4 T cells (Fig. 5c) but very striking for CD8 T cells (Fig. 5d) [34]. In young patients, TBI mainly affected CD4 T cell subsets, indeed in these patients we observed a reduction of naïve T cells and the increase of EM CD4 T cells (Fig. 5c), and these changes did not show a relation to injury severity (Fig. 5e). Instead, in CD8 T cells, young TBI patients had a slight, not significant, reduction of naïve CD8 T cells, and overall, no changes in CM, EM, and EMRA CD8 T cells were observed (Fig. 5d). In elderly TBI patients, we observed a statistically significant reduction of naïve T cells accompanied by the increase of CD4 CM T cells (Fig. 5c), and the increase of CD4 CM T cells was more evident in severe patients (Fig. 5e). Moreover, we observed a statistically significant increase of CD8 CM (Fig. 5d) (in mild elderly TBI patients, Fig. 5f) and EM (moderate TBI patients), and the reduction of EMRA T cells (mild elderly TBI patients). In summary, these data showed that trauma activated lymphocytes which indeed present different stages of T-cell differentiation compared to control subjects. However, while young patients mainly present a phenotype indicative of a differentiation process of CD4 T cells, in elderly patients both CD4 and especially CD8 T cells were affected by TBI.

**Fig. 6:**
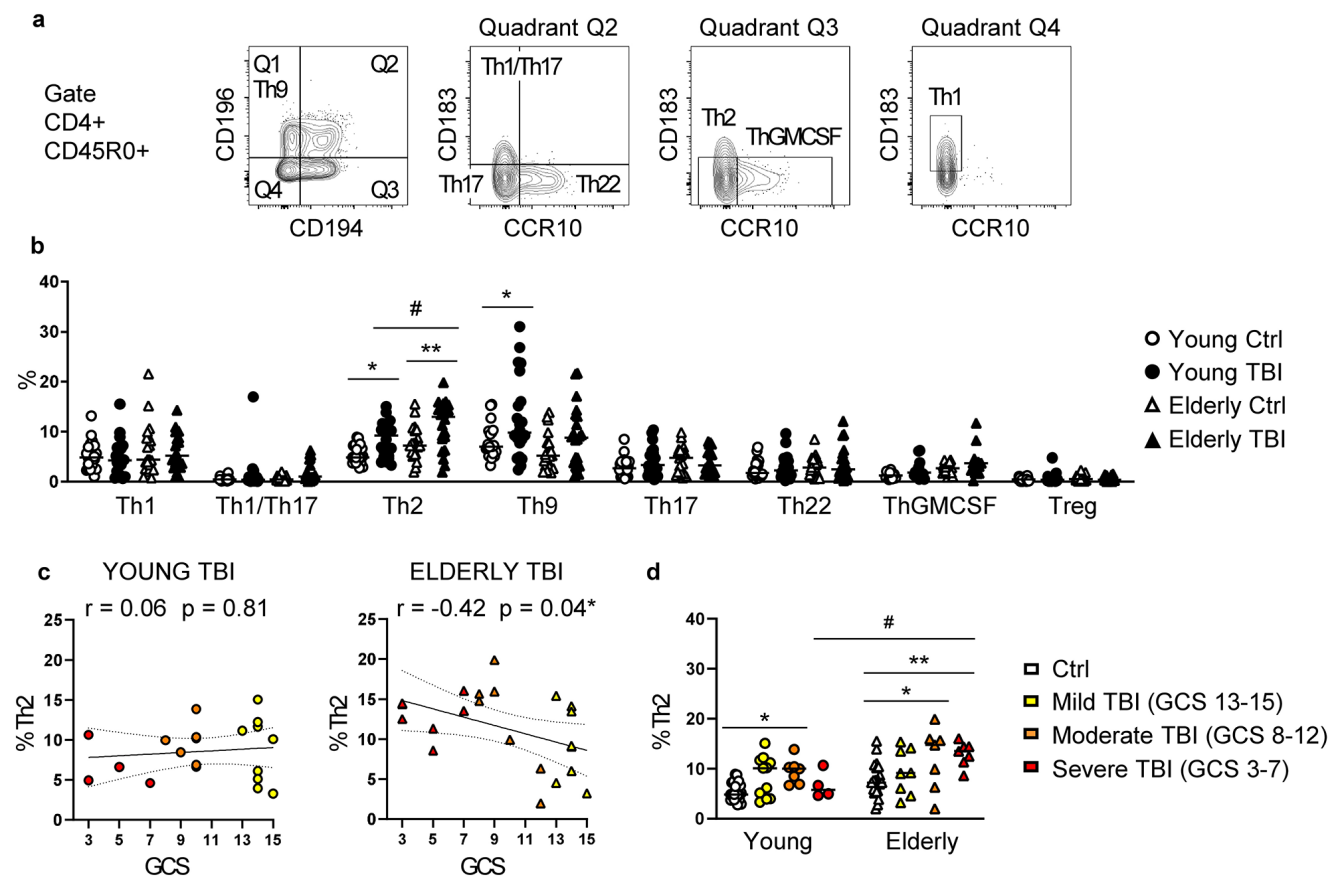
Analysis of CD4 Th subsets. (**a**) Gating strategy used to identify Th subsets: Th subsets were identified in the CD3 + CD4 + CD45RA- CD45R0 + gate, according to the expression of CD183/CXCR3, CD194/CCR4, CD196/CCR6, and CCR10 as follows: Th1 (CD194- CD196- CD183+ CCR10-), Th2 (CD194+ CD196- CD183- CCR10-), Th17 (CD194+ CD196+ CD183- CCR10-), Th1/Th17 (CD194+ CD196+ CD183+ CCR10-), Th22 (CD194+ CD196+ CD183- CCR10+), Th9 (CCR4- CCR6+), and ThGM-CSF (CCR4+ CCR6- CXCR3- CCR10+). Regulatory T cells (Treg) were identified as CD25hiFoxP3+. (**b**) Percentage of Th subsets is shown in PBMC from young (circles) and elderly (triangles) donors, control (white symbols) and TBI patients (black symbols). (**c**) Correlation between GCS score and the percentages of Th2 in young and elderly TBI patients. Correlations were assessed by Spearman correlation coefficients, and dashed lines represent 95% confidence intervals. (**d**) Percentages of Th2 subset are shown according to injury severity. * p < 0.05; ** p < 0.01 TBI vs. control aged-matched subjects. # p < 0.05 young vs. elderly TBI patients

Once primed, naïve CD4 T cells proliferate and differentiate into multiple heterogeneous subsets, with distinct effector (mainly helper) functions. Therefore, we next investigated how TBI affects the main T helper (Th) subsets (i.e., both classical Th cells, such as the Th1, Th2, and Th17 subsets, and non-classical Th1/Th17, Th22, Th9, ThGM-CSF), and regulatory T cells (Tregs). Among all the CD4 T cell subsets investigated, TBI significantly increased the Th9 subset in young patients, and the Th2 subset in both young and elderly patients, with a greater effect in elderly compared to young TBI patients (Fig. 6b). Differently from young TBI patients, elderly ones showed an injury-dependent effect. In fact, correlation analysis showed that in elderly TBI patients lower GCS scores aligned (p = < 0.05) with higher percentages of Th2 cells (Fig. 6c), and, as a confirmation, the significant increase of Th2% in elderly TBI patients was observed in severe and moderate (and not in mild) TBI patients (Fig. 6d).

**Fig. 7:**
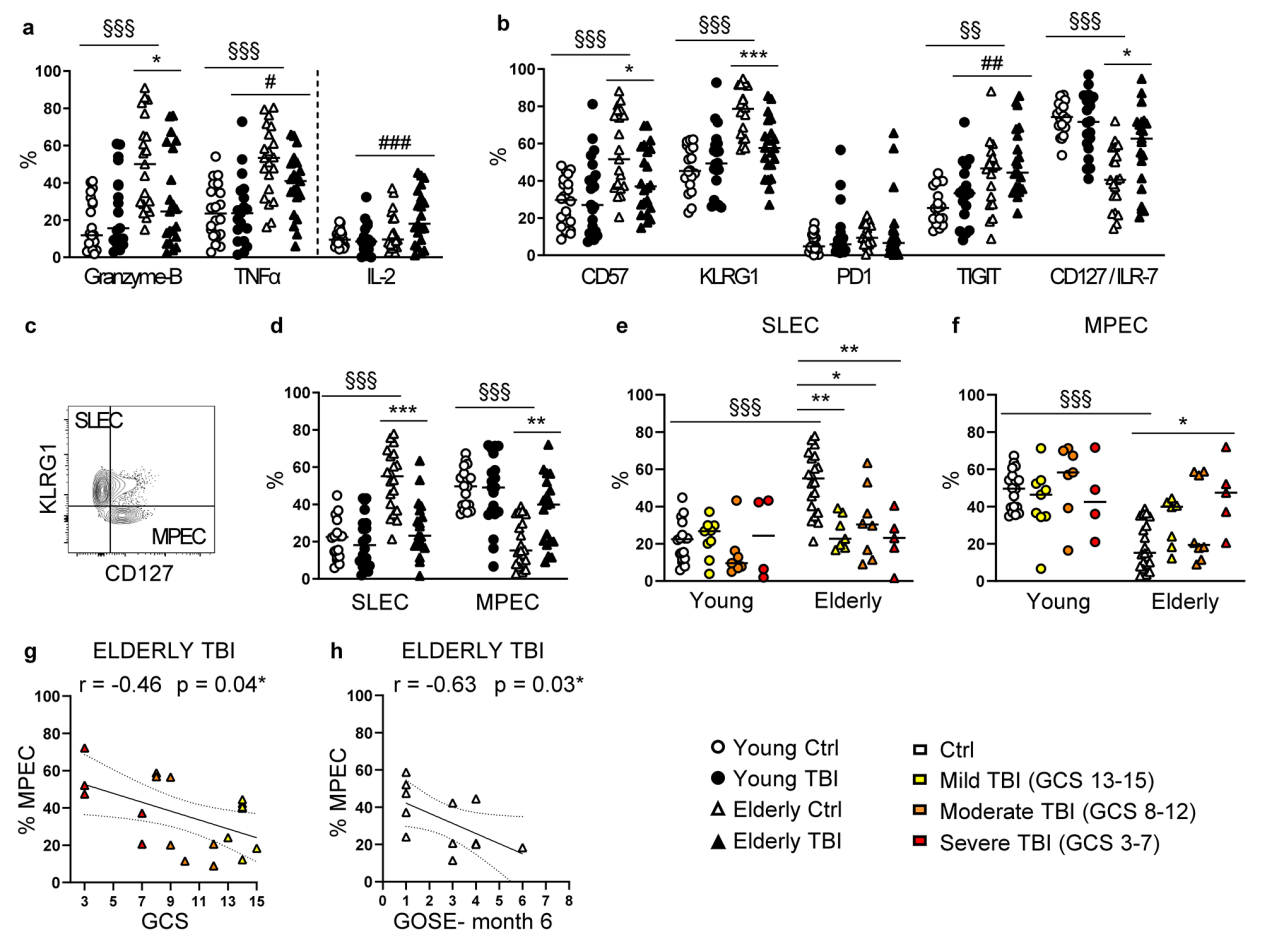
Analysis of CD8 T cell differentiation. (**a**) Intracellular expression of Granzyme B, TNF-α, and IL-2 in CD8 T cells (CD3+ CD4- T cells) after PMA/Ionomycin stimulation for 3 h. (**b**) Percentage of expression of CD57, KLRG1, PD1, TIGIT, CD127/ILR-7 on CD8 T cells. (**c**) Within CD8 T cells, short-lived effector cells (SLEC) and memory precursor effector cells (MPEC) were identified as KLRG1+ CD127-, and KLRG- CD127+, respectively. (**d**) Percentage of SLEC and MPEC CD8 T cells. Results were shown in PBMC from young (circles) and elderly (triangles) donors, control (white symbols) and TBI patients (black symbols). (**e**) Percentage of SLEC, and (**f**) of MPEC CD8 T cells, was also shown separating patients according to injury severity assessed by GCS score. In elderly TBI patients, the injury severity (GCS score, panel **g**) and the 6-month outcome (GOSE value, panel **h**) were correlated (Spearman correlation) with the percentage of MPEC cells. * p < 0.05; ** p < 0.01; *** p < 0.001 TBI vs. control aged-matched subjects. §§ p < 0.01; §§§ p < 0.001 young vs. elderly control subjects. # p < 0.05; ## p < 0.01; ### p < 0.001 young vs. elderly TBI patients

CD8 T cells are generally recognized as cytotoxic cells, very effective in the direct lysis of infected target cells, and characterized by the expression of cytotoxic molecules and pro-inflammatory cytokines such as Granzyme B and TNF-α. As reported by other studies, our results confirmed the secretion of high levels of Granzyme B and TNF-α in elderly compared to young controls (Fig. 7a), a typical feature of aged T cells that indeed presents the so-called senescence-associated secretory phenotype (SASP), leading to inflammaging [35]. TBI induced a significant reduction in expression of both Granzyme B and TNF-α in elderly TBI patients, while no differences were observed in young patients (Fig. 7a), reinforcing the notion that TBI affects CD8 T cells only in the elderly.

Since a limited cytotoxic capacity and cytokine production observed in elderly TBI patients could be indicative of a progressive loss of effector function known as cell exhaustion, we evaluated also the expression of IL-2 considering that IL-2 production is one of the first functions to be lost in exhausted CD8 T cells [36]. Contrary to expected, we observed an increased production of IL-2 in elderly TBI patients (Fig. 7a). To better understand these results, we investigated the expression of other markers usually associated with a senescent or exhausted cell phenotype (i.e., CD57, KLRG1, PD1, TIGIT, CD127). As expected, CD8 T cells from elderly controls showed higher percentages of CD57, KLRG1, TIGIT, and a lower expression of IL-7 receptor (CD127) with respect to young control subjects (Fig. 7b). Again, the phenotype of young TBI patients did not differ from that of aged-matched control subjects. Instead, in elderly patients, TBI reduced the expression of CD57 and KLRG1 and increased the expression of CD127 (Fig. 7b). Overall, these findings exclude that the limited cytotoxic capacity of CD8 T cells from elderly TBI patients can be explained with a CD8 exhaustion phenotype, but suggest that PBMC from elderly patients may have altered CD8 T cell functions (in part due to different inflammatory cytokine secretion, Fig. 7a) or could be in a specific stage of T cell differentiation.

KLRG1, together with CD127, that were significantly altered in elderly TBI patients, have been largely used as markers to identify at least two effector cell subsets that have different fates and memory cell developmental potential. Specifically, they have been used to identify the short-lived effector cells (SLEC, CD8 effector T cells with robust, but short-term cytotoxic function), and the memory precursor effector cells (MPEC, that can become long-lived memory CD8 T cells) [37]. We therefore analyzed the frequency of SLEC, defined by the expression of KLRG1 (KLRG1+ CD127-), and of MPEC, which retain the expression of CD127 (KLRG- CD127+) (Fig. 7c). In healthy controls, aging resulted in a significant increment of SLEC and a decrease of MPEC (Fig. 7d). TBI did not change SLEC and MPEC frequency in young subjects, while it reversed the age-related alterations observed in elderly controls as shown by a significant decrease of SLEC and increase of MPEC subsets (Fig. 7d). Interestingly, in elderly patients, the reduction of SLEC did not show a relation to injury severity (Fig. 7e), while the increase of MPEC was statistically significant for severe TBI patients (Fig. 7f). Furthermore, in elderly TBI patients, lower GCS scores were significantly associated with a higher frequency of MPEC (Fig. 7g), and the higher the frequency of MPEC, the worse the long-term outcome (Fig. 7h).

### Alterations in B lymphocyte subsets

Considering their key role of mediators of immunity, we next investigated how TBI affects the frequency of different CD19+ B cell subsets in both young and elderly groups.

**Fig. 8:**
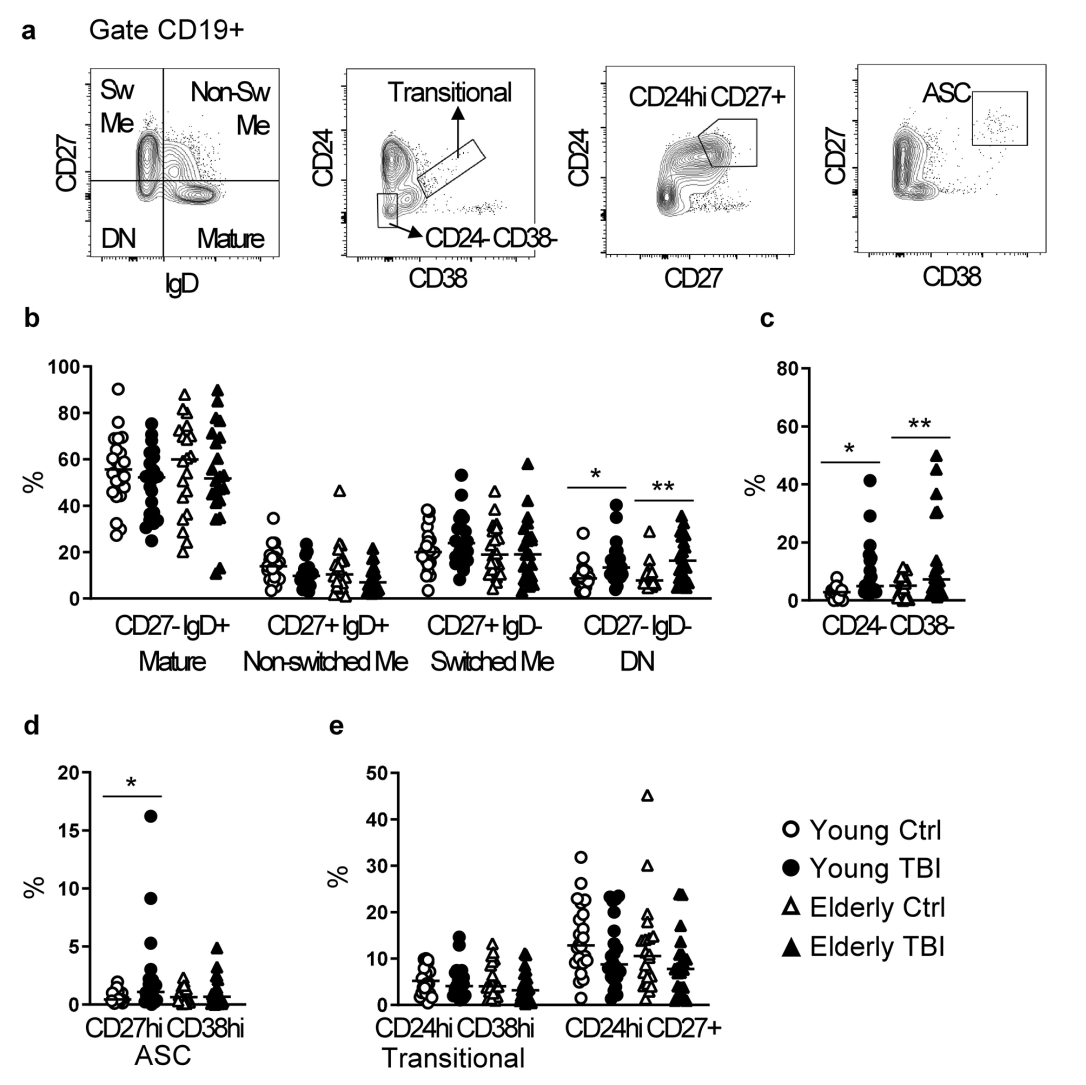
Analysis of B lymphocyte subsets. (**a**) Gating strategy used to identify B cell subsets, in the gate CD19+, and based on the expression of CD27 and IgD, or CD24 and CD38, or CD24 and CD27, or CD27 and CD38. (**b**) Percentage of mature (IgD+ CD27-), non-switched memory (IgD+ CD27+), switched memory (IgD- CD27+), and double-negative B cells (IgD- CD27-). (**c**) Percentage of CD24- CD38- B cells. (**d**) Percentage of antibody secreting cells (ASC, CD27hi CD38hi)). (**e**) Percentage of regulatory B cells evaluated as CD24hi CD38hi (transitional B cells) and CD24hi CD27+. Analyses were performed in PBMC from young (circles) and elderly (triangles) donors, control (white symbols) and TBI patients (black symbols). * p < 0.05; ** p < 0.01 TBI vs. control aged-matched subjects

First, we analyzed four core CD19+ B-cell subsets based on the expression of CD27 and IgD, specifically mature (CD27- IgD+), non-switched memory (CD27+ IgD+), switched memory (CD27+ IgD-), and double-negative (CD27- IgD-) B cells [38] (Fig. 8a). TBI induced some alterations in the B cell compartment, and these changes did not show a relation to injury severity (data not shown). After TBI, double-negative CD27- IgD- subset increased in both young and elderly TBI patients (Fig. 8b).

Double-negative B cells represent a heterogenous subset of memory B cells, and within memory B cells, a particular memory subset defined as CD38- CD24- (Fig. 8a) has been described to be enriched in elderly people [39]. TBI increased the frequency of CD38- CD24- memory B cell subset in both young and elderly patients (Fig. 8c).

Next, since previous studies provided evidence that TBI is associated with temporal changes in humoral immunity (changes in serum levels of IgG and IgM) [11, 14], we analyzed the percentage of antibody secreting cells (ASC) identified as CD27hi CD38hi cells (Fig. 8a). Compared to aged-matched control subjects, TBI induced a small, but statistically significant, increase of ASC in young patients (Fig. 8d).

Finally, we analyzed two different B cell populations with regulatory functions: transitional CD38hi CD24hi B cells, and CD24hi CD27 + B cells [40] (Fig. 8a). The frequency of both Breg populations did not change after TBI in both young and elderly patients (Fig. 8e).

### Alterations in NK cell subsets

**Fig. 9:**
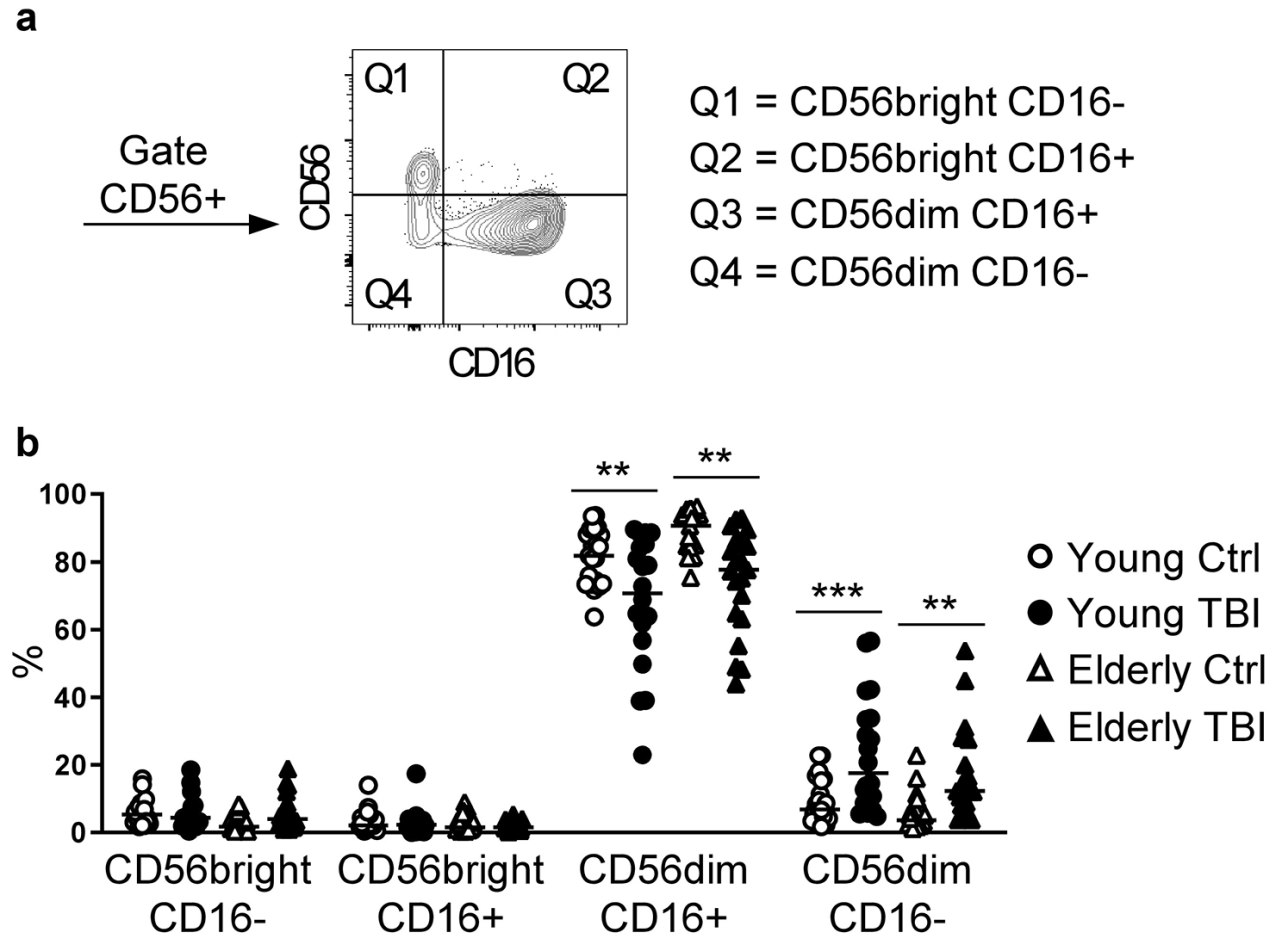
Analysis of NK cell subsets. (**a**) Gating strategy applied to identify NK cell subsets, in the gate CD56+, and based on the expression of CD56 and CD16. (**b**) Percentage of NK subsets in PBMC from young (circles) and elderly (triangles) donors, control (white symbols) and TBI patients (black symbols). ** p < 0.01; *** p < 0.001 TBI vs. control aged-matched subjects

NK cells can be classified into several subsets according to the expression of adhesion molecule CD56 and of FcγRIIIA receptor CD16. The majority of peripheral blood NK cells expresses low levels of CD56, are positive for CD16 (CD56dim CD16+), and present high cytotoxic activity. The second NK cell subset present in the peripheral blood expresses high levels of CD56 and is negative for CD16 (CD56bright CD16-), and is mostly involved in the production of cytokines [41]. Peripheral blood furthermore contains a low frequency of CD56dim CD16- and CD56bright CD16+ NK cells [42]. Thus, in PBMC from young and elderly TBI patients and aged-matched control subjects, we evaluated the percentage of the four CD56+ NK cell subsets (Fig. 9a). After TBI, we did not find any differences in CD56bright CD16– and CD56bright CD16+ subsets in both patient cohorts, while we observed a significant reduction of CD56dim CD16+ and an increase of the CD56dim CD16- subset, both in young and elderly patients (Fig. 9b). These alterations did not show a relation to injury severity (data not shown).

### Alterations in monocyte subsets

**Fig. 10:**
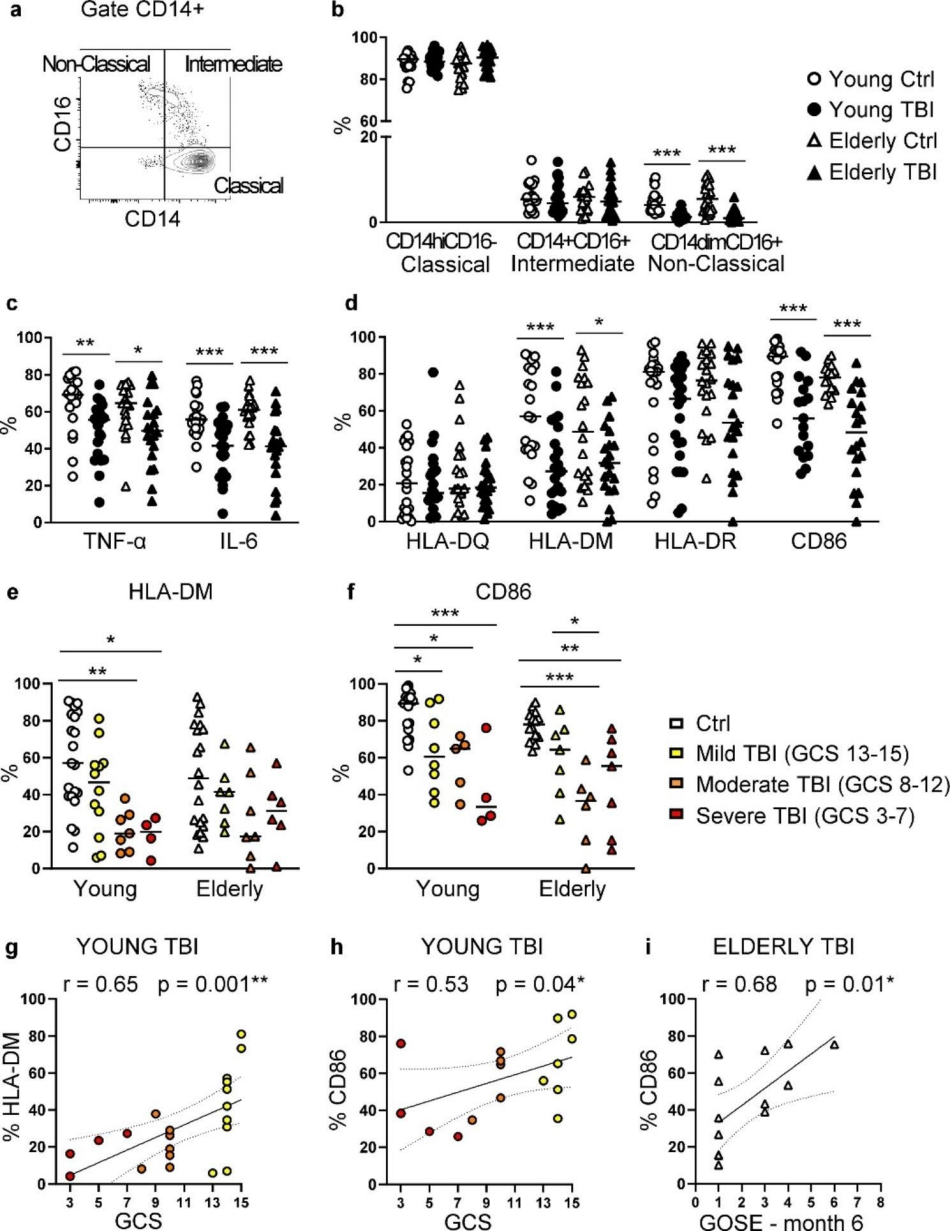
Analysis of monocyte subsets and activation. (**a**) Gating strategy applied to identify monocyte cell subsets, in the gate CD14+, and based on the expression of CD16 and CD14. (**b**) Percentage of classical (CD14hi CD16-), intermediate (CD14+ CD16+), non-classical (CD14dim CD16+) monocytes. (**c**) Intracellular expression of TNF-α, and IL-6 in monocytes after LPS stimulation for 5.5 h. (**d**) Percentage of expression of HLA-DQ, HLA-DM, HLA-DR, and CD86 on monocytes. Analyses were performed in PBMC from young (circles) and elderly (triangles) donors, control (white symbols) and TBI patients (black symbols). (**e**) Percentage of HLA-DM, and (**f**) of CD86 on monocytes are also shown separating patients according to injury severity assessed by GCS score. (**g**) In young TBI patients, the injury severity (GCS score) was correlated (Spearman correlation) with the percentage of HLA-DM. (**h**) In elderly TBI patients, the injury severity (GCS score) and (**i**) the 6-month outcome (GOSE value) were correlated (Spearman correlation) with the percentage of CD86. * p < 0.05; ** p < 0.01; *** p < 0.001 TBI vs. control aged-matched subjects

Human monocytes have been classified into three subtypes based on relative surface expression of LPS co-receptor CD14 and FCγ III receptor CD16. Classical monocytes, the most abundant monocyte subset, are strongly positive for CD14 and do not express CD16 (CD14^hi^CD16-), non-classical monocytes have very low expression of CD14 and high levels of CD16 (CD14^dim^CD16+), and intermediate monocytes express both markers (CD14 + CD16+) [43] (Fig. 10a). TBI significantly reduced the non-classical monocytes CD14^dim^CD16 + subsets, in both young and elderly patients (Fig. 10b).

To investigate the impact of TBI on immunological function of monocytes, we analyzed the intracellular expression of the pro-inflammatory cytokines TNF-α and IL-6. After TBI, monocytes from both young and elderly TBI patients produced significantly reduced levels of TNF-α and IL-6 (Fig. 10c).

Due to their roles in driving monocytes to efficiently act as antigen presenting cells and to patrol innate immune responses, we next examined the expression of human leukocyte antigen (HLA) class II (i.e. HLA-DQ, -DM, -DR) in addition to the co-stimulatory molecules CD86/B7-2. Despite some heterogeneity due to the individual variability of each subject, our data showed an altered phenotype of CD14+ monocytes of both young and elderly TBI patients characterized by a reduced expression of HLA-DM, HLA-DR, and CD86, compared to respective age-matched control subjects (Fig. 10d). In young TBI patients, the reduction of HLA-DM and CD86 was injury-dependent, as demonstrated by data analysis for injury severity (Fig. 10e-f), and by correlation analysis with GSC score (Fig. 10g-h). In elderly TBI patients, a worse long-term outcome is associated with a greater reduction of CD86 (Fig. 10i).

## Discussion

In this study we depicted the global peripheral immune landscape in the acute phase of TBI in young and aged patients. A strength of this study is that elderly patients are compared to aged-matched control subjects, therefore alterations observed are very likely TBI specific, and not attributable to the functional immune senescence that PBMC undergo with age. At first, we described TBI patient features in relation to age and, as expected, while enrolled young and elderly patients showed similar distribution among injury TBI severities (as GCS score post stabilization) their outcome was dramatically divergent, with elderly population showing higher mortality and worse long-term outcome, in accordance with previous independent cohorts [22, 44–46]. Plasmatic biomarkers related to neuronal (NfL and Tau) and astrocytic/BBB (GFAP) damage were significantly increased after TBI, with an injury-severity dependency, reflecting results obtained from larger patient cohorts [19, 47]. Of note, an age-related effect was observed for NfL levels, which was higher in both elderly controls and TBI patients, indicating that TBI in elderly population results in additive susceptibility to neuronal damage. In addition, we evidenced a strong inflammatory condition in TBI patients as shown by the increased levels of IL-6 and IL-10 in TBI serum, with an injury-severity dependency, in line with literature [19, 48]. Moreover, both cytokines were higher in elderly TBI patients compared to younger TBI patients, indicating an amplification of systemic immune reaction associated with age.

We then analysed the age effect on immune cell activation after TBI, and we demonstrated that immune cells from young and elderly TBI patients respond differently to TBI. Indeed, adaptive immune cells (T and B lymphocytes) of elderly patients were more affected by TBI, while innate immune cells (NK and monocytes) showed no major age-related differences after TBI. Interestingly, we identified new subsets that to our knowledge have not been previously described and which were increased in TBI, namely memory precursor effector (MPEC) CD8 T cells, CD27- IgD- and CD38- CD24- B cells, and cytotoxic CD56- CD16- NK cells, and we defined the subsets that are differently expressed in elderly patients. Moreover, we defined which alterations are injury-related.

Specifically, our results outlined that TBI alters the systemic levels of T cells and age affects their ability to become activated and to differentiate. Although the lower viability of PBMC from TBI patients could have induced a bias on the analyzed cells, we showed a significant reduction in the frequencies of T cells in agreement with literature [12–15, 29, 49]. This T cell decrease was observed in both young and elderly patients. TBI reduced the frequency of CD4 and increased that of CD8 T cells, and the CD4/CD8 ratio revealed a lower ratio after TBI, as previously reported in animal models [50, 51]. These alterations reached statistical significance mainly in severe patients. It is important to acknowledge that a low CD4/CD8 ratio was considered an immune risk phenotype and was associated with the risk for chronic inflammation, frailty, age-related disease, and poor prognosis in different diseases such as systemic lupus erythematosus, metastatic cancer, diabetes and myocardial infarction [52–55]. Furthermore, elderly TBI patients show increased levels of both activated (CD45R0+) CD4 and CD8 T cells, while young TBI patients presented an increase mainly of activated CD4 T cells. This might be plausible considering that age-associated alterations in the epigenome are different between CD4 and CD8 T cells, making CD8 T cells more susceptible to activation [56]. In addition, we observed that age affects the differentiation of TBI-derived T cells, indeed we observed that CD4 T cells from young patients preferentially differentiate toward effector memory (EM) cells, while those from elderly patients mainly differentiate toward central memory (CM) T cells. Of note, T EM cells have a lower proliferative capacity but display high cytotoxicity; in contrast, CM cells have a high proliferative capacity but do not produce microbicidal cytokines, and thus exhibit low cytotoxicity [57].

In parallel, we found an imbalance in effector CD4 T cell differentiation in TBI patients. More specifically, there was an increased frequency of Th9 subset in young and increased Th2 cell subset in both young and elderly patients, with respect to other T cell subsets (Th1, Th17, Th1/Th17, Th22, ThGM-CSF, and Treg). Th9 cells have an unknown role in TBI, they produce IL-9, which was reported high in plasma of TBI patients [58]. The increase of Th2 herein observed also confirms previous studies indicating an imbalance towards Th2 due to the overproduction of the Th2-type cytokine IL-4 by PBMC [28, 59] or detected in the serum [60, 61] of TBI patients. Interestingly, in elderly TBI patients, the Th2 increase was injury related, with a significant increase of the Th2 subset in severe and moderate TBI subjects. Of note, Th2 T cells produce cytokines that inhibit and counteract phagocytic macrophage functions and Th1-mediated response [62], suggesting a possible role of Th2 T cells in the TBI-induced immunodepression [63, 64] leading to a poor outcome, mainly observed in elderly patients.

TBI also impacts the differentiation ability of CD8 T cells, generally recognized as cytolytic cells. In contrast to CD8 T cells from young patients which maintain a differentiation ability similar to healthy control subjects, CD8 T cells from elderly patients show increased differentiation of both CM and EM cells with a concomitant increase of IL-2 production, which could sustain their expansion [33]. In addition, CD8 T cells from elderly TBI patients were skewed towards the memory MPEC (KLRG- CD127+) subset, differently from control subjects that instead presented a higher percentage of SLEC (KLRG1+ CD127-). MPEC, that have not been previously investigated in TBI patients, are effector T cells with the ability to differentiate into multiple memory cell lineages, including CM and EM [37], supporting the observed enrichment of both CM and EM CD8 T cells in elderly patients. More importantly, MPEC are less efficient compared to SLEC in producing inflammatory cytokines and cytotoxic molecules [37]. We indeed observed reduced Granzyme B and TNF-α production in CD8 T cells from elderly TBI patients, compared to aged-matched control subjects. Interestingly, in the elderly the increase of MPEC frequency is significant only in severe TBI patients, suggesting a role of this immune alteration in driving the severity of the trauma. Furthermore, elderly patients with a higher MPEC frequency seem to have worse outcomes, making this cell population a possible novel prognostic marker or an attractive immune target in TBI, that needs further investigations. Collectively, our data highlighted the immune unfavourable status of T cells from elderly patients compared to young patients. Indeed, in elderly patients both CD4 and CD8 T cells are affected by TBI and are induced to differentiate into subtypes with low cytotoxic activity, thus posing additional challenges, mainly in the case of infections.

Similar to T lymphocytes, B cell differentiation was also affected by trauma. Previous studies provided evidence of several B-cell associated alterations in TBI, however the B-cell contribution to the injury still remains unclear. Alterations in serum levels of IgG and IgM [14] and in autoantibody production have been described in some individuals [11], and these findings are indirectly supported by our data which evidence increased levels of antibody secreting cells (ASC), mainly in young patients. Autoantibody production can persist for many years, and has been associated with poor patient outcome [11]. For the first time, our study shows an increased frequency of two B cell subsets in both young and elderly TBI patients that have never been investigated in TBI until now. Specifically, we observed an increase of the CD38- CD24- subset, described to produce TNF-α and to be likely involved in inflammaging in the elderly [39], and a concomitant increase of the CD27- IgD- B cell subset, also known as double negative (DN) B cells. This represents a unique population of memory B cells contributing to the pathogenesis of autoimmune diseases, including systemic lupus erythematosus, myasthenia gravis, multiple sclerosis, and rheumatoid arthritis [65, 66]. In these pathologies DN B cells have the ability to differentiate into plasma cells and to produce high levels of proinflammatory cytokines such as TNF-α, lymphotoxin-α, and Granzyme B [66]. Therefore, both CD38- CD24- and DN B cell subset, might contribute to the peripheral exacerbated inflammatory reaction which plays a relevant role in secondary TBI injury [67]. However, DN B cells were found enriched also in infectious diseases such as malaria, HIV infection and rotavirus infection [68–70]. Differently from what observed in autoimmune diseases, during infection DN B cells expressed characteristics of premature exhaustion, with high levels of inhibitory receptor and poor proliferation in response to stimulation, leading to defective responses against infection [66]. Therefore, an understanding of the functionality of B cells might be important to elucidate their role in TBI, and evaluation of B cell cytokine production, in order to assess their pro-inflammatory status or on the contrary, their low-cytotoxic status as observed for T cells, need further investigation.

In contrast to the adaptive immune populations, TBI alters the innate immune cell populations (monocytes and NK cells) similarly in both elderly and young patients, with very few significant changes specifically linked to age.

Monocytes, together with neutrophils, are the major immune cell population that are activated immediately after trauma [18], and infiltrate into the damaged brain tissue post-injury [71]. In line with other human studies [15, 18, 72, 73], we observed a great increase of the frequency of peripheral monocytes. However, there are very few studies on the different monocyte subsets involved in TBI. Here, in line with a recent study conducted on young severe TBI patients [74], we showed a significant reduction of the non-classical monocyte CD14dim CD16+ subset in both young and elderly TBI patients. In contrast with Janicova’s study [74], we did not detect changes in either the intermediate CD14+ CD16+ or classical CD14hi CD16- monocyte populations. The different type of sample (fresh whole blood vs. thawed PBMC), and the different score of injury (severe TBI vs. severe, moderate, and mild TBI), could account for the differences observed between the study performed by Janicova and colleagues [74] and our investigation. Also, the different sample timing (within the first 12 h post-injury vs. a media of 21 h) was shown to be a fundamental aspect when considering immune trauma-induced changes [18]. The reduction of non-classical monocytes herein observed could suggest their specific recruitment into the brain. Importantly, the requirement of non-classical monocytes for the infiltration of neutrophils into the brain, and the critical role of non-classical monocytes in mediating secondary injury and neurocognitive outcome was demonstrated in animal models [9].

In addition, our data showed a reduced expression of HLA-DM, HLA-DR and of co-stimulatory molecule CD86/B7-2 in monocytes from both young and elderly TBI patients, suggesting that TBI compromises monocyte ability to efficiently act as antigen presenting cells. The presence of CD14+ HLA-DRlow/− monocytes with reduced expression of CD86 was previously observed in the early phases post injury [18], and the expression of HLA-DR antigen in monocytes has been correlated with the clinical course of patients, identifying patients with low HLA-DR expression as patients at high risk of infection and death following trauma [75]. Interestingly, in young patients we found an injury-severity relationship with the low expression of HLA-DM or CD86 on monocytes. In addition, in elderly TBI patients a worse outcome seems to be associated with lower CD86+ monocytes, suggesting their role in disease progression. Moreover, after LPS stimulation monocytes expressed less TNF-α and IL-6 pro-inflammatory cytokines compared to healthy controls, in line with a previous report [18]. TNF-α and IL-6 are blood biomarkers that are increased after TBI [6], and we indeed confirm the increased level of IL-6 in the plasma of TBI patients. The fact that herein we found that stimulated monocytes produce less IL-6 and TNF-α could indicate that TBI have induced an exhausted state of monocytes [76], with a reduced ability to drive a pro-inflammatory response, thus contributing to post-injury complications, such as infections. On the other hand, the elevated pro-inflammatory cytokines found in the blood could derive from other cells, such as from injured brain cells (i.e. microglia and astrocytes) [77], from circulating neutrophils [78], one of the first responders activated immediately after TBI and that persist for days after trauma [18], or from endothelium cells who can become activated within minutes from trauma [78]. Monocytes recruited into the brain could differentiate into neuroinflammatory or neuroprotective macrophages that play an important role in the evolution of TBI. As a matter of fact, monocyte depletion was shown to improve functional outcomes in a TBI experimental model [9]. It was also reported that macrophage response to TBI initially involves heterogeneous polarization with simultaneous mixed M1/M2 phenotype generated due to the complex signaling events surrounding them [79–81]. The altered phenotype of monocytes observed could be indicative of a commitment of monocytes towards a less inflammatory, M2-like phenotype, however further studies are needed to better address this issue.

As previously reported [14, 82], we observed a decreased frequency of CD56+ NK cells, but only in elderly TBI patients, and a lower percentage of NK cells was reported in infected patients with severe TBI compared to non-infected patients [14]. When we analysed the different NK subsets, we observed a reduction of the CD56dim CD16+ subset similar in both young and elderly patients, suggesting that the age of patients did not affect the reactivity of the major cytotoxic NK sub-population, at least in the acute phase after TBI. On the other hand, we found a significant increase of the CD56dim CD16- population, never described before in TBI patients. CD16 is quickly shed from the NK cell surface upon activation due to the action of metalloproteases [83], and therefore the highest percentage of degranulating and cytotoxic NK cells was reported to be among the CD56dim CD16- subset [84]. In previous studies, based only on the analysis of the intensity expression of CD56, an increase of perforin-expressing cells within CD56dim NK cells on 1 day after injury was described [30]. The CD56dim CD16- subset, that here we observed increased in TBI patients, might therefore represent the principal perforin-expressing NK cell subset previously reported. Since perforin-expressing NK cells have been positively correlated with lower appearance of infections in severe TBI [30], further studies to dissect the cytotoxic role of CD56dim CD16+ and CD56dim CD16- are needed. Overall, also NK cells represent one of the immune cell populations rapidly activated post-TBI, with a particular increase of the cytotoxic CD56dim CD16- subset.

## Conclusions

Our study elucidates the acute immune cell aberrations in patients following TBI. We describe a different starting immune status in young and elderly subjects, and more important, a different immune response after trauma in young and elderly TBI patients, which could drive the severity of the injury and, possibly, the outcome. Specifically, while in young only CD4 T cells are activated by TBI, in elderly both CD4 and CD8 T cells are affected, and are induced to differentiate into subtypes with low cytotoxic activity, such as CM CD4 T cells and MPEC CD8 T cells, underlining the immune unfavourable status of elderly patients compared with young ones. In addition, we identify specific subsets of B cells induced by TBI (CD27- IgD- and CD38- CD24-), whose role in the disease needs further investigation. Cells belonging to the innate immunity showed a similar magnitude of activation between different ages, whereby we report an induction of CD56dim CD16- NK cells and an increase of monocytes with compromised ability to drive a pro-inflammatory response and to efficiently act as antigen presenting cells. The results presented herein help better clarify the pathophysiology of TBI, and could help establish which immune cells could become biomarkers of disease prognosis and/or potential therapeutic targets for individualised medicine. Further studies are needed to investigate the immune cell alterations that occur not only in the acute phase, as performed in this study, but also in subacute and chronic phase after TBI, in order to provide new insights on the post-traumatic evolution of the injury and response to therapy. Moreover, in order to decipher brain versus general-injury impacts, other control groups could be investigated, such as orthopedic trauma group [85, 86].

## Methods

### Subject recruitment

A total of 45 TBI patients admitted to the Fondazione IRCCS Ca’ Granda Ospedale Maggiore Policlinico of Milan (Italy) were included in this study. Patient characteristics are listed in Table 1. Blood samples from TBI patients were collected within 48 h from injury, after obtaining informed written consent.

A total of 22 young (18-45yo) and 21 elderly (> 65yo) control subjects (enrolled by Fondazione Poliambulanza of Brescia, Italy and Istituto di Ricerche Farmacologiche Mario Negri IRCCS of Milano, Italy) were included in the study. The collection of blood samples was performed after obtaining informed written consent and data were fully anonymized for the study.

Exclusion criteria for both TBI patients and healthy control subjects included infections, immunological disorders, immunosuppressive medication, pregnancy or nursing, and previous neurological disorders which could confound the evaluation of TBI related neurological sequelae.

### Isolation of plasma and biomarker analysis

Bloods were collected in EDTA tubes, centrifuged 10 min at 2000 rcf, RT and plasma samples were aliquoted and cryopreserved at -80° for biomarker analyses. Levels of NfL, total Tau, GFAP, IL-6 and IL-10 were measured using commercially available single molecule array assay kits on an SR-X Analyzer (NfL: #103,186, total Tau #103,806, GFAP #102,336, IL-6 # 101,622 and IL-10 #101,643 advantage kits) as described by the manufacturer (Quanterix, Billerica, MA). A single batch of reagents was used for each analyte. Samples were diluted 1:200 in diluent buffer. A single lot of reagents was used for all samples.

### Isolation of PBMC

Peripheral blood mononuclear cells (PBMC) were separated from sodium citrate whole blood through density gradient centrifugation (Histopaque, Sigma-Aldrich or BD Vacutainer CPT Mononuclear Cell Preparation Tube, BD Biosciences), frozen in different aliquots at the concentration of 1–5 millions/mL in fetal bovine serum (FBS, Sigma-Aldrich) with 10% DMSO (Sigma-Aldrich), and stored in liquid nitrogen.

### Flow cytometry analysis of PBMC subsets

Cryopreserved PBMC were thawed and counted with Trypan blue and then stained to determine the different cellular subsets, according to standard staining procedures. First, to exclude dead cells from analyses PBMC were stained with eBioscience™ Fixable Viability Dye eFluor™ 780 (Thermo Fisher Scientific, Waltham, MA USA), according to the manufacturer’s instructions. Then, PBMC subsets were identified by staining with pre-determined dilutions of fluorochrome-conjugated anti-human antibodies for 20 min at 4 °C (See Supplementary Tables 3, Additional File 3). For markers which needed intracellular staining, the Fixation/Permeabilization Solution Kit (BD) was used. Stain buffer used for washing and antibodies incubations consisted of 0.02% sodium azide and 0.1% bovine serum albumin in PBS (Sigma-Aldrich). Cells were acquired on FACSAria III or FACSymphony A3 calibrated with CST beads before each experiment (all from BD Biosciences). Electronic compensation was performed using single-stained controls prepared with control PBMC and antibody-capture beads (BD Biosciences). Data were acquired with the FACSDiva V 8.0 software (BD) and analyzed using FlowJo 10.8 software (Treestar, LLC, USA).

### Flow cytometry analysis of PBMC cytokine production

After thawing, PBMC were incubated at 37 °C and 5% CO2 in RPMI 1640 medium supplemented with 10% FBS, 1% penicillin/ streptomycin, and 2 mM L-glutamine and stimulated to induce cytokine production. Specifically, to analyse the intracellular expression of Granzyme B, TNF-α, and IL-2 by T cells, PBMC were stimulated for a total of 3 h with phorbol 12-myristate 13-acetate (PMA, 10 ng/ml) and ionomycin (500 ng/ml) (both from Sigma-Aldrich) in the presence of the protein transport inhibitors GolgiPlus (brefeldin A, 1 µl/ml, BD Biosciences) added after 1 h. Instead, to analyse the intracellular expression of IL-6 and TNF-α by monocytes, PBMC were stimulated for a total of 5.5 h with lipopolysaccharide (LPS, 100 ng/ml, Sigma-Aldrich) in the presence of GolgiPlus (brefeldin A, 1 µl/ml, BD Biosciences) added after 1 h.

After stimulation, PBMC were washed in PBS and then stained with eBioscience™ Fixable Viability Dye eFluor™ 780 (Thermo Fisher Scientific, Waltham, MA USA) according to the manufacturer’s instructions to exclude dead cells. T cells or monocytes were identified by cell surface staining with specific fluorochrome-conjugated anti-human antibodies (20 min at 4 °C) followed by intracellular staining of cytokines (30 min at 4 °C) (See Supplementary Tables 3, Additional File 3) performed with Fixation/Permeabilization Solution Kit (BD Biosciences). Cells were acquired on FACSAria III or FACSymphony A3 calibrated with CST beads before each experiment (all from BD Biosciences). Electronic compensation was performed using single-stained controls prepared with control PBMC and antibody-capture beads (BD Biosciences). Data were acquired with the FACSDiva V 8.0 software (BD) and analyzed using FlowJo 10.8 software (Treestar, LLC, USA).

### Statistical analysis

Two-way analysis of variance (ANOVA), with main effects age (young vs. elderly), group (control vs. TBI) and corresponding interaction effect age x group, was used. Tukey’s correction post hoc tests were applied for multiple comparison. Correlation between GCS or GOSE values and the percentage of each parameter evaluated was performed using the Spearman rank correlation coefficient. P < 0.05 was considered to be statistically significant. Statistical analyses were performed using GraphPad Prism software 9 (GraphPad Software, La Jolla, CA, USA).

### Electronic supplementary material

Below is the link to the electronic supplementary material.

**Supplementary Table 1**. Blood biomarkers

**Supplementary Table 2**. PBMC characterization

**Supplementary Table 3**. List of monoclonal antibodies used in the study.

## Data Availability

All data generated or analysed during this study are included in this published article [and its supplementary information files].

## Abbreviations

TBI: Traumatic brain injury
PBMC: Peripheral blood mononuclear cells
NK: Natural killer
NfL: Neurofilament light
GFAP: Glial fibrillary acidic protein
