# Supplementary material for: Systemic immune response in young and elderly patients after traumatic brain injury
Source: Immun Ageing. 2023 Aug 12;20:41. doi: 10.1186/s12979-023-00369-1 (PMC10422735; doi:10.1186/s12979-023-00369-1)
Supplement: Supplementary file 1 — Supplementary Table 1. Blood biomarkers [file 12979_2023_369_MOESM1_ESM.docx]

**Supplementary Table 1. Blood biomarkers**

| **Biomarker** | **Young Ctrl**  Log10 value  (Median, IQR) | **Young TBI**  Log10 value  (Median, IQR) | **Elderly Ctrl**  Log10 value  (Median, IQR) | **Elderly TBI**  Log10 value  (Median, IQR) | **Injury**  **effect**  p value | **Age effect**    p value | **Injury x Age Interaction** p value | **Young Ctrl**  **vs**  **Young TBI**  p value | **Elderly Ctrl**  **vs**  **Elderly TBI**  p value | **Young Ctrl**  **vs**  **Elderly Ctrl**  p value | **Young TBI**  **vs**  **Elderly TBI**  p value |
| --- | --- | --- | --- | --- | --- | --- | --- | --- | --- | --- | --- |
| NFL | 0.52 (0.46; 0.75) | 1.38 (1.29; 1.83) | 1.27 (1.03; 1.38) | 1.80 (1.47; 1.90) | <0.0001 | 0.0005 | ns | <0.0001 | 0.0079 | 0.0090 | 0.0264 |
| Tau | 0.02 (-0.17; 0.31) | 0.66 (0.30; 0.98) | -0.18 (-0.36; 0.05) | 0.64 (0.39; 1.35) | 0.0002 | ns | ns | 0.0410 | 0.0013 | ns | ns |
| GFAP | 1.51 (1.44; 1.69) | 3.94 (3.20; 4.13) | 2.21 (2.14; 2.55) | 4.02 (3.11; 4.65) | <0.0001 | 0.0628 | ns | <0.0001 | <0.0001 | ns | ns |
| IL-6 | -0.27 (-0.32; 0.13) | 1.27 (1.03; 1.72) | 0.16 (-0.10; 0.19) | 1.80 (1.38; 2.11) | <0.0001 | 0.0310 | ns | <0.0001 | <0.0001 | ns | 0.0237 |
| IL-10 | 0.57 (0.53; 0.70) | 1.00 (0.90; 1.27) | 0.48 (0.39; 0.51) | 1.31 (1.19; 1.51) | <0.0001 | ns | 0.0057 | <0.0001 | <0.0001 | ns | 0.0059 |
