# Supplementary material for: Systemic immune response in young and elderly patients after traumatic brain injury
Source: Immun Ageing. 2023 Aug 12;20:41. doi: 10.1186/s12979-023-00369-1 (PMC10422735; doi:10.1186/s12979-023-00369-1)
Supplement: Supplementary file 2 — Supplementary Table 2. PBMC characterization [file 12979_2023_369_MOESM2_ESM.docx]

**Supplementary Table 2. PBMC characterization**

| **Immune Population** | **Young Ctrl**  %  Median (IQR) | **Young TBI**  %  Median (IQR) | **Elderly Ctrl**  %  Median (IQR) | **Elderly TBI**  %  Median (IQR) | **Injury**  **effect**  p value | **Age effect**  p value | **Injury X Age Interaction**  p value | **Young Ctrl vs Young TBI**  p value | **Old Ctrl vs Elderly TBI**  p value | **Young Ctrl vs Elderly Ctrl**  p value | **Young TBI vs**  **Elderly TBI**  p value |
| --- | --- | --- | --- | --- | --- | --- | --- | --- | --- | --- | --- |
| **T lymphocytes (CD3+)** | 68.2 (64.4; 71.5) | 40.1 (29.9; 57.5) | 54.9 (42.9; 67.3) | 38.7 (31.0; 46.2) | <0.0001 | 0.0005 | ns | <0.0001 | <0.0001 | 0.0028 | ns |
| **CD4+** | 63.1 (53.3; 71.2) | 52.4 (43.6; 67.4) | 72.1 (60.0; 82.4) | 59.5 (50.2; 74.3) | 0.0066 | 0.0017 | ns | ns | ns | 0.0468 | 0.0499 |
| CD4+CD45R0+ | 33.8 (28.5; 42.2) | 49.0 (38.5; 65.5) | 41.6 (32.7; 51.8) | 55.7 (36.7; 69.6) | <0.0001 | ns | ns | 0.0013 | 0.0260 | ns | ns |
| CD4 Naive (CD45RA+CD197+) | 57.3 (45.6; 64.3) | 32.6 (23.5; 55.1) | 46.1 (36.3; 54.3) | 31.0 (16.2; 45.6) | 0.0001 | 0.0252 | ns | 0.0021 | 0.0482 | ns | ns |
| CD4 Central Memory (CD45RA-CD197+) | 27.4 (20.6; 32.7) | 29.2 (24.3; 44.8) | 31.1 (23.6; 38.2) | 43.0 (32.0; 51.0) | 0.0088 | 0.0090 | ns | ns | 0.0205 | ns | 0.0229 |
| CD4 Effector Memory (CD45RA-CD197-) | 10.9 (7.51; 15.9) | 23.2 (12.4; 33.1) | 14.3 (9.34; 20.0) | 18.0 (10.8; 31.0) | 0.0004 | ns | ns | 0.0012 | ns | ns | ns |
| CD4 Effector Memory RA+ (CD45RA+CD197-) | 2.92 (0.79; 5.78) | 2.62 (0.89; 8.97) | 5.68 (3.34; 8.32) | 1.66 (0.73; 4.17) | ns | ns | 0.0354 | ns | ns | ns | ns |
| Th1 | 4.82 (2.56; 7.00) | 4.26 (1.86; 6.66) | 4.42 (2.51; 8.90) | 5.21 (3.04; 9.09) | ns | ns | ns | ns | ns | ns | ns |
| Th1/Th17 | 0.43 (0.33; 0.62) | 0.39 (0.19; 0.85) | 0.47 (0.22; 0.87) | 1.02 (0.58; 3.60) | 0.0102 | ns | ns | ns | ns | ns | ns |
| Th2 | 4.85 (3.88; 6.82) | 9.21 (5.08; 10.8) | 7.22 (5.52; 10.3) | 13.0 (8.02; 14.9) | <0.0001 | 0.0008 | ns | 0.0106 | 0.0019 | ns | 0.0136 |
| Th9 | 6.98 (5.59; 9.90) | 9.80 (7.04; 17.6) | 5.21 (3.16; 7.76) | 8.78 (3.49; 13.1) | 0.0025 | 0.0221 | ns | 0.0142 | ns | ns | ns |
| Th17 | 2.66 (1.09; 3.96) | 3.34 (1.64; 5.83) | 4.76 (1.62; 6.26) | 3.24 (1.81; 5.92) | ns | ns | ns | ns | ns | ns | ns |
| Th22 | 1.77 (1.19; 3.58) | 2.25 (1.26; 4.17) | 2.86 (1.34; 4.52) | 2.46 (1.47; 4.85) | ns | ns | ns | ns | ns | ns | ns |
| ThGM-CSF | 1.26 (0.70; 1.88) | 1.80 (1.10; 3.36) | 2.70 (1.73; 3.33) | 3.66 (1.76; 3.95) | 0.0171 | 0.0045 | ns | ns | ns | ns | ns |
| Treg | 0.46 (0.10; 0.74) | 0.34 (1.16; 0.66) | 0.51 (0.27; 0.86) | 0.41 (0.21; 0.79) | ns | ns | ns | ns | ns | ns | ns |
| **CD3+CD8+** | 34.7 (28.6; 46.5) | 47.5 (32.3; 56.0) | 27.3 (17.3; 38.3) | 39.7 (25.2; 49.5) | 0.0068 | 0.0017 | ns | ns | ns | 0.0484 | 0.0475 |
| CD8+CD45R0+ | 26.0 (17.6; 31.0) | 32.4 (25.0; 38.3) | 34.2 (24.9; 51.4) | 48.2 (37.8; 60.7) | 0.0328 | <0.0001 | ns | ns | ns | 0.0012 | 0.0006 |
| CD8 Naive (CD45RA+CD197+) | 44.8 (34.3; 59.0) | 36.1 (26.1; 48.7) | 13.5 (4.43; 24.0) | 12.2 (6.61; 25.9) | 0.0253 | <0.0001 | ns | ns | ns | 0.0012 | 0.0011 |
| CD8 Central Memory (CD45RA-CD197+) | 7.33 (4.77; 8.91) | 4.92 (4.14; 12.1) | 5.65 (3.02; 10.7) | 14.0 (8.64; 20.8) | 0.0083 | 0.0037 | 0.0430 | ns | 0.0057 | ns | 0.0035 |
| CD8 Effector Memory (CD45RA-CD197-) | 21.4 (14.7; 29.9) | 25.5 (23.0; 31.7) | 20.9 (14.5; 30.7) | 34.3 (20.0; 45.7) | 0.0047 | ns | ns | ns | 0.0074 | ns | ns |
| CD8 Effector Memory RA+ (CD45RA+CD197-) | 25.5 (14.9; 34.6) | 25.5 (12.0; 41.2) | 51.8 (43.0; 72.4) | 26.0 (20.5; 45.4) | ns | <0.0001 | 0.0029 | ns | 0.0032 | <0.0001 | ns |
| SLEC (KLRG1+CD127-) | 22.5 (11.9; 26.4) | 18.2 (7.21; 29.9) | 55.1 (37.7; 66.9) | 23.1 (16.8; 36.9) | 0.0001 | <0.0001 | 0.0003 | ns | <0.0001 | <0.0001 | ns |
| MPEC (KLRG1-CD127+) | 49.7 (38.9; 57.6) | 49.0 (35.1; 65.1) | 15.3 (8.18; 33.5) | 39.9 (18.3; 52.1) | 0.0372 | <0.0001 | 0.0113 | ns | 0.0074 | <0.0001 | ns |
| Granzyme-B | 11.8 (4.33; 30.1) | 15.6 (9.25; 39.0) | 50.0 (27.7; 71.3) | 24.6 (9.43; 60.4) | ns | <0.0001 | 0.0045 | ns | 0.0291 | <0.0001 | ns |
| TNF-α | 23.5 (11.9; 38.8) | 23.7 (8.70; 35.7) | 53.3 (34.1; 68.2) | 41.0 (27.6; 50.7) | ns | <0.0001 | ns | ns | ns | <0.0001 | 0.0238 |
| IL-2 | 9.43 (6.07; 12.9) | 8.48 (4.3; 15.6) | 9.55 (6.60; 21.1) | 18.0 (7.94; 34.1) | ns | 0.0011 | ns | ns | ns | ns | 0.0010 |
| CD57 | 29.7 (14.8; 38.8) | 27.0 (12.4; 45.8) | 51.6 (36.8; 76.6) | 37.0 (24.8; 57.4) | ns | <0.0001 | 0.0158 | ns | 0.0438 | <0.0001 | ns |
| KLRG1 | 45.3 (37.0; 58.3) | 49.3 (32.3; 59.8) | 78.7 (63.3; 91.5) | 57.6 (44.9; 69.1) | 0.0187 | <0.0001 | 0.0008 | ns | 0.0004 | <0.0001 | ns |
| PD1 | 4.71 (3.16; 11.1) | 5.91 (2.56; 10.7) | 9.37 (6.38; 16.7) | 6.64 (3.45; 13.6) | ns | ns | ns | ns | ns | ns | ns |
| TIGIT | 25.4 (18.9; 37.9) | 33.3 (17.7; 46.1) | 46.7 (27.3; 53.3) | 44.4 (35.6; 66.1) | ns | <0.0001 | ns | ns | ns | 0.0078 | 0.0039 |
| CD127/IL7R | 74.2 (67.6; 81.6) | 71.6 (58.1; 84.5) | 40.5 (25.1; 56.8) | 62.7 (38.5; 71.7) | ns | <0.0001 | 0.0107 | ns | 0.0129 | <0.0001 | ns |
| **CD4/CD8** **ratio** | 1.84 (1.15; 2.49) | 1.10 (0.78; 2.09) | 2.64 (1.58; 4.84) | 1.50 (1.01; 2.95) | ns | 0.0007 | ns | ns | ns | 0.0087 | ns |
| **B lymphocytes (CD19+)** | 8.19 (5.76; 9.47) | 7.7 (4.9; 9.6) | 6.06 (3.33; 8.71) | 7.55 (4.18; 10.1) | ns | ns | ns | ns | ns | ns | ns |
| Mature (CD27-IgD+) | 55.6 (45.4; 65.4) | 52.2 (35.7; 61.4) | 59.9 (38.2; 71.9) | 51.7 (42.2; 69.9) | ns | ns | ns | ns | ns | ns | ns |
| Non-switched (CD27+IgD+) | 13.9 (9.05; 18.0) | 9.74 (5.93; 12.7) | 10.4 (5.63; 16.3) | 6.97 (3.69; 12.1) | 0.0068 | ns | ns | ns | ns | ns | ns |
| Switched (CD27+IgD-) | 20.0 (15.2; 25.6) | 23.9 (16.9; 31.4) | 18.9 (11.1; 30.1) | 19.0 (9.34; 27.5) | ns | ns | ns | ns | ns | ns | ns |
| Double Negative (CD27-IgD-) | 8.74 (6.91; 10.8) | 13.4 (10.3; 18.0) | 7.81 (6.61; 11.6) | 16.3 (8.46; 27.0) | <0.0001 | ns | ns | 0.0278 | 0.0026 | ns | ns |
| CD24-CD38- | 2.87 (1.10; 3.51) | 4.94 (2.93; 14.0) | 5.06 (1.44; 7.92) | 7.27 (3.62; 17.9) | 0.0003 | ns | ns | 0.0386 | 0.0073 | ns | ns |
| ASC (CD27hi CD38hi) | 0.44 (0.25; 0.99) | 1.08 (0.47; 2.13) | 0.63 (0.33; 1.51) | 0.67 (0.09; 1.59) | 0.0288 | ns | ns | 0.0106 | ns | ns | ns |
| Transitional (CD38hi CD24hi) | 5.18 (2.46; 7.96) | 4.05 (2.60; 6.80) | 4.04 (2.65; 9.20) | 3.18 (1.27; 6.04) | ns | ns | ns | ns | ns | ns | ns |
| CD24hiCD27+ | 12.8 (9.09; 20.1) | 8.73 (5.94; 17.0) | 10.6 (4.94; 14.7) | 7.74 (3.33; 10.9) | 0.0378 | ns | ns | ns | ns | ns | ns |
| **NK cells (CD56+)** | 9.34 (4.91; 11.0) | 5.29 (2.96; 8.49) | 14.7 (9.48; 21.5) | 6.94 (3.21; 11.3) | 0.0003 | 0.0005 | 0.0168 | ns | 0.0002 | 0.0003 | ns |
| CD56brightCD16- | 5.27 (2.81; 7.54) | 4.37 (2.22; 7.90) | 1.63 (0.91; 3.66) | 3.88 (1.92; 8.12) | ns | ns | ns | ns | ns | ns | ns |
| CD56brightCD16+ | 2.13 (1.37; 3.96) | 2.20 (0.34; 3.37) | 1.48 (0.85; 4.47) | 1.49 (1.16; 2.50) | ns | ns | ns | ns | ns | ns | ns |
| CD56dimCD16+ | 81.5 (73.5; 88.5) | 70.8 (58.1; 85.0) | 90.6 (84.6; 94.8) | 78.9 (64.4; 87.2) | <0.0001 | 0.0197 | ns | 0.0043 | 0.0011 | ns | ns |
| CD56dimCD16- | 6.80 (4.62; 15.5) | 17.6 (8.82; 33.7) | 3.54 (2.45; 6.00) | 12.2 (8.51; 27.8) | <0.0001 | ns | ns | 0.0007 | 0.0018 | ns | ns |
| **Monocytes (CD14+)** | 11.4 (8.21; 15.6) | 34.6 (25.2; 48.4) | 13.1 (8.59; 21.5) | 41.3 (37.0; 50.3) | <0.0001 | ns | ns | <0.0001 | <0.0001 | ns | ns |
| Classical (CD14hi CD16-) | 89.5 (86.1; 90.4) | 88.46 (86.0; 92.5) | 87.6 (81.5; 90.6) | 90.3 (84.9; 93.7) | 0.0341 | ns | ns | ns | ns | ns | ns |
| Intermediate (CD14+ CD16+) | 5.36 (3.52; 8.86) | 4.46 (2.97; 8.36) | 5.93 (2.87; 7.02) | 4.82 (1.99; 7.35) | ns | ns | ns | ns | ns | ns | ns |
| Non classical (CD14dim CD16+) | 4.01 (2.71; 6.05) | 1.24 (0.79; 2.10) | 5.48 (2.66; 8.78) | 0.90 (0.41; 1.83) | <0.0001 | ns | ns | <0.0001 | <0.0001 | ns | ns |
| HLA-DQ+ | 20.8 (4.80; 42.2) | 15.6 (11.5; 27.8) | 17.9 (10.9; 36.0) | 18.5 (9.89; 27.3) | ns | ns | ns | ns | ns | ns | ns |
| HLA-DM+ | 57.0 (39.0; 84.0) | 27.3 (15.5; 51.3) | 48.7 (23.2; 77.2) | 31.8 (18.0; 47.2) | <0.0001 | ns | ns | 0.0008 | 0.0458 | ns | ns |
| HLA-DR+ | 81.0 (41.8; 85.0) | 66.4 (35.8; 81.8) | 76.5 (62.2; 90.0) | 53.6 (31.8; 81.4) | 0.0233 | ns | ns | ns | ns | ns | ns |
| CD86+ | 89.4 (72.5; 93.9) | 56.0 (36.9; 73.9) | 78.0 (70.5; 83.4) | 48.3 (28.6; 68.7) | <0.0001 | ns | ns | 0.0001 | <0.0001 | ns | ns |
| TNF-α | 69.1 (59.5; 76.9) | 55.6 (35.5; 61.4) | 64.6 (54.1; 73.0) | 49.8 (37.8; 60.4) | 0.0001 | ns | ns | 0.0044 | 0.0203 | ns | ns |
| IL-6 | 55.8 (49.7; 63.1) | 41.7 (26.7; 51.5) | 61.0 (55.5; 64.1) | 41.2 (28.3; 50.0) | <0.0001 | ns | ns | 0.0007 | <0.0001 | ns | ns |
