# Supplementary material for: Systemic immune response in young and elderly patients after traumatic brain injury
Source: Immun Ageing. 2023 Aug 12;20:41. doi: 10.1186/s12979-023-00369-1 (PMC10422735; doi:10.1186/s12979-023-00369-1)
Supplement: Supplementary file 3 — Supplementary Table 3. List of monoclonal antibodies used in the study. [file 12979_2023_369_MOESM3_ESM.docx]

**Supplementary Table 3. List of monoclonal antibodies used in the study.**

| **Specifity** | **Fluorocrome** | **Clone** | **Manufacturer** | **Cat. #** | **Titer used** (µL) |  |
| --- | --- | --- | --- | --- | --- | --- |
| **CD3** | APC | UCHT1 | BD Biosciences | 555335 | 2 | |
| **CD3** | BV510 | UCHT1 | BD Biosciences | 563109 | 0.7 | |
| **CD4** | BB700 | RPA-T4 | BD Biosciences | 745981 | 0.25 | |
| **CD4** | FITC | SK3 | BD Biosciences | 566911 | 0.25 | |
| **CD8** | APC | RPA-T8 | BD Biosciences | 555369 | 1 | |
| **CD11b** | BB700 | ICRF44 (44) | BD Biosciences | 742210 | 0.7 | |
| **CD14** | A647 | MφP9 | BD Biosciences | 562690 | 0.5 | |
| **CD14** | BV510 | MΦP9 | BD Biosciences | 563079 | 0.5 | |
| **CD16** | PE | 3G8 | BD Biosciences | 555407 | 1.7 | |
| **CD19** | BB700 | SJ25C1 | BD Biosciences | 566396 | 0.5 | |
| **CD24** | BV421 | ML5 | BD Biosciences | 562789 | 0.5 | |
| **CD25** | BV421 | M-A251 | BD Biosciences | 562442 | 0.5 | |
| **CD27** | PE-Cy7 | M-T271 | BD Biosciences | 560609 | 0.7 | |
| **CD38** | PE | HB7 | BD Biosciences | 345806 | 0.7 | |
| **CD45RA** | PerCP Cy5.5 | HI100 | BD Biosciences | 563429 | 1 | |
| **CD45RO** | PE-CF594 | UCHL1 | BD Biosciences | 562299 | 0.7 | |
| **CD56** | PE-Vio770 | REA196 | Miltenyi | 130-113-313 | 0.25 | |
| **CD57** | PE-CF594 | NK-1 | BD Biosciences | 562488 | 0.05 | |
| **CD86** | PE-CF594 | 2331 (FUN-1) | BD Biosciences | 562390 | 1 | |
| **CD127** | PE-Vio770 | MB15-18C9 | Miltenyi | 130-113-412 | 1 | |
| **CD138** | APC | 44F9 | Miltenyi | 130-117-395 | 0.3 | |
| **CD183/CXCR3** | PE | 1C6/CXCR3 | BD Biosciences | 557185 | 2.5 | |
| **CD194/CCR4** | PE-Vio770 | REA279 | Miltenyi | 130-118-359 | 1 | |
| **CD196/CCR6** | BV421 | 11A9 | BD Biosciences | 562515 | 1 | |
| **CD197** | BV421 | 150503 | BD Biosciences | 562555 | 2.5 | |
| **CD279/PD-1** | PE | MIH4 | BD Biosciences | 557946 | 5 | |
| **CCR10** | APC | 1B5 | BD Biosciences | 564771 | 0.3 | |
| **FOXP3** | PE-CF594 | 259D/C7 | BD Biosciences | 562421 | 2.5 | |
| **HLA-DM** | PE | MaP.DM1 | BD Biosciences | 555983 | 2 | |
| **HLA-DQ** | FITC | TU169 | BD Biosciences | 555563 | 2 | |
| **HLA-DR** | PE-Cy7 | G46-6 | BD Biosciences | 560651 | 0.7 | |
| **IgD** | FITC | IA6-2 | BD Biosciences | 555778 | 1 | |
| **KLRG1** | PE-Vio615 | REA261 | Miltenyi | 130-120-427 | 1 | |
| **TIGIT** | BV421 | 741182 | BD Biosciences | 747844 | 0.25 | |
| **Granzyme B** | PE-CF594 | GB11 | BD Biosciences | 562462 | 0.6 | |
| **IL-2** | PE | MQ1-17H12 | BD Biosciences | 554566 | 1 | |
| **IL-6** | FITC | MQ2-13A5 | BD Biosciences | 554544 | 2 | |
| **TNFa** | FITC | MAb11 | BD Biosciences | 554512 | 0.5 | |
| **TNFa** | PE-Cy7 | MAb11 | BD Biosciences | 557647 | 2 | |
